# Simultaneous Overexpression of Functional Human HO-1, E5NT and ENTPD1 Protects Murine Fibroblasts against TNF-α-Induced Injury *In Vitro*

**DOI:** 10.1371/journal.pone.0141933

**Published:** 2015-10-29

**Authors:** Alessandro Cinti, Marco De Giorgi, Elisa Chisci, Claudia Arena, Gloria Galimberti, Laura Farina, Cristina Bugarin, Ilaria Rivolta, Giuseppe Gaipa, Ryszard Tom Smolenski, Maria Grazia Cerrito, Marialuisa Lavitrano, Roberto Giovannoni

**Affiliations:** 1 Department of Surgery and Translational Medicine, University of Milano-Bicocca, Monza, Italy; 2 Medical University of Gdansk, Gdansk, Poland; 3 M. Tettamanti Research Center, Pediatric Clinic, University of Milano Bicocca, Monza, Italy; 4 Department of Health Sciences, University of Milano-Bicocca, Monza, Italy; University of Alabama at Birmingham, UNITED STATES

## Abstract

Several biomedical applications, such as xenotransplantation, require multiple genes simultaneously expressed in eukaryotic cells. Advances in genetic engineering technologies have led to the development of efficient polycistronic vectors based on the use of the 2A self-processing oligopeptide. The aim of this work was to evaluate the protective effects of the simultaneous expression of a novel combination of anti-inflammatory human genes, ENTPD1, E5NT and HO-1, in eukaryotic cells. We produced an F2A system-based multicistronic construct to express three human proteins in NIH3T3 cells exposed to an inflammatory stimulus represented by tumor necrosis factor alpha (TNF-α), a pro-inflammatory cytokine which plays an important role during inflammation, cell proliferation, differentiation and apoptosis and in the inflammatory response during ischemia/reperfusion injury in several organ transplantation settings. The protective effects against TNF-α-induced cytotoxicity and cell death, mediated by HO-1, ENTPD1 and E5NT genes were better observed in cells expressing the combination of genes as compared to cells expressing each single gene and the effect was further improved by administrating enzymatic substrates of the human genes to the cells. Moreover, a gene expression analyses demonstrated that the expression of the three genes has a role in modulating key regulators of TNF-α signalling pathway, namely *Nemo* and *Tnfaip3*, that promoted pro-survival phenotype in TNF-α injured cells. These results could provide new insights in the research of protective mechanisms in transplantation settings.

## Introduction

The expression of multiple proteins in eukaryotic cells has become crucial in many biomedical applications of contemporary cell biology [1]. Advances in genetic engineering technologies have led to the production and development of efficient polycistronic vectors essentially based on two strategies: the use of internal ribosome entry site (IRES) sequence [1] or the 2A self-processing oligopeptide [2,3]. The 2A coexpression system works in all eukaryotic expression systems and in all cell types [2] and it is based on the co-translationally self-processing activity of 2A sequences, such that each constituent protein encoded by a single mRNA is generated as a discrete translation product [4]. The 2A and ‘2A-like’ sequences have been successfully used to express several proteins in lentivirus-mediated gene therapy approaches [5] and in the production of monoclonal antibodies in transgenic mice [6].

Another biomedical application requiring multiple genetic modifications in eukaryotic cells is xenotransplantation, where the complexity of immunological barriers to be overcome for a successful experiment requires several human genes to be overexpressed in the cells of the potential donor species [7,8]. In pig to non-human primates models two main processes, hyperacute rejection (HAR) and acute vascular rejection (AVR), rapidly attack vascularized organs [9]. Transplanted organs are also subjected to several antigen-independent injuries, such as ischemia/reperfusion injury (IRI) [10,11] and free radicals production [12].

Over the years several genes, whose induction or over-expression is able to modulate the inflammatory response and preserve the metabolism of transplanted organs were identified.

Heme oxygenase 1 (HO-1) plays a protective role by preventing oxidative stress because of its antioxidant and antiapoptotic properties and via suppression of the immune response through anti-inflammatory mechanisms [13–15]. In a model of cardiopulmonary by-pass, we previously demonstrated the protective effects of HO-1/CO against ischemia reperfusion injury [16]. Anyhow transgenic expression of hHO-1 is promising to prolong survival of xenografts but should be part of multiple transgenic modification for xenotransplantation.

Vascular endothelium within the transplanted organ is the primary target of rejection for all the mechanisms described in xenotransplantation. The nucleotide metabolism of endothelial cells may contribute significantly to the vascular diseases in acute humoral rejection [17]. Nucleosides and catabolites of adenosine in mammals are of particular interest in the field of xenotransplantation due to their combined cytoprotective, immunosuppressive and anti-inflammatory effects [18–20]. Extracellular adenosine is produced by a pathway mediated mainly by ectonucleotidases ecto-nucleoside triphosphate diphosphohydrolase 1 (ENTPD1 or CD39), and ecto-5’-nucleotidase (E5NT or CD73) [21]. Several *in-vitro* or *in-vivo* models have been produced with genetic defects or overexpression of ENTPD1 or E5NT [22–25] to investigate the role of these proteins in modulating inflammation. The aim of this work was to evaluate the protective effects of the simultaneous expression of a novel combination of anti-inflammatory human genes, ENTPD1, E5NT and HO-1, in eukaryotic cells. We produced an F2A system-based multicistronic construct to express three human proteins in murine NIH3T3 cells exposed to an inflammatory stimulus represented by human tumor necrosis factor alpha (TNF-α), a pro-inflammatory cytokine which plays an important role in the immune system during inflammation, cell proliferation, differentiation and apoptosis [26] and in the inflammatory response during ischemia/reperfusion injury in several organ transplantation settings [8,27–30].

This study demonstrated, for the first time, the protection against inflammatory stimuli of a novel combination of human genes, when they are simultaneously expressed in murine NIH3T3 cells.

## Material and Methods

### Reagents and antibodies

Recombinant human TNF-α, hemin and ATP were purchased from Sigma Aldrich. NIH3T3 cells were treated with reagents diluted in complete medium at concentrations determined by preliminary experiments and detailed below. Anti-hE5NT (4G4, Novus Biologicals), anti-hHO-1 (EP1391Y, Epitomics) and anti-hENTPD1 (BU61, Santa Cruz) primary antibodies, Alexa Fluor 488-conjugated anti-mouse and Alexa Fluor 555-conjugated anti-rabbit (Life Technologies) secondary antibodies were used for immunofluorescence analysis. Anti-hHO-1 (EP1391Y, Epitomics), anti-hE5NT (EPR6115, LifeSpan BioSciences), anti-hENTPD1 (HPA014067, Sigma Aldrich), anti-β-actin (AC-15, Sigma Aldrich)), anti-Nf-kB1 p105/p50 (D4P4D, Cell Signaling) and anti-Lamin B2 (EPR9701(B), Abcam) primary antibodies were used for immunoblotting analysis. Phycoerythrin (PE)-conjugated anti-hE5NT (BD Biosciences) and Alexa Fluor 647-conjugated anti-hENTPD1 (Life Technologies) antibodies were used for FACS analysis and cell sorting.

### Triple cistronic vector construction

The triple cistronic vector was prepared following a strategy similar to those previously reported by Ryan *et al*. [31] and by our group [32]. Briefly, the coding sequence of the plasmid pcDNA3.1-hHO1-F2A1-hE5NT-F2A2-hENPTD1 [32] was excised and ligated into pCX-C1 plasmid (a pCX-EGFP plasmid [33] to which a neomycin resistance cassette has been added) to form the final pCX-hHO1-F2A1-hE5NT-F2A2-hENTPD1-C1, which was called pCX-TRI-2A. Control plasmids, expressing only one of the three human proteins at once, were prepared by ligation of the PCR-amplified coding sequence into pCX-C1 plasmid, to obtain pCX-hHO1, pCX-hE5NT and pCX-hENTPD1 respectively. To facilitate cell sorting of pCX-hHO1-transfected cells, EGFP gene was cloned in frame downstream the HO1/F2A sequence. Restriction and sequencing analyses were performed on all the intermediate and in the final construct. Empty pCX-C1 plasmid was used for mock transfections.

### Cell culture and transfection

NIH3T3 cells were grown in Dulbecco’s minimum essential medium (DMEM) (EuroClone) supplemented with 10% fetal calf serum (Sigma Aldrich), at 37°C and 5% CO_2_.

Cells were split and plated to reach 80–90% confluence on the day of transfection. pCX-TRI-2A, pCX-HO1, pCX-hE5NT, pCX-hENTPD1 and empty vector plasmids were introduced into NIH3T3 cells by electroporation using Neon Transfection System (Life Technologies) according to the manufacturer’s instructions for NIH3T3 cell type. Cells were immediately re-suspended in growth medium with serum without antibiotics and plated. After 24 hours, cells were transferred in standard medium plus 0.5 mg/ml of G418 (Sigma Aldrich) and selected for 7 days.

### Flow Cytometry analysis and cell sorting

WT and pCX-TRI-2A transfected cells were detached with trypsin/EDTA and washed once with PFN buffer (serum 3%, NaN_3_ 0,01% in PBS). Cells were then incubated for 30 min in the dark with fluorophore-conjugated anti-hE5NT and anti-hENTPD1 antibodies. The excess and non-specifically bound antibodies were removed by washing with PFN buffer. Flow cytometric analysis of stained cells was performed with a FACSAria flow cytometer (Becton Dickinson). Lymphocytes were used as a positive control, wild type and mock-transfected NIH3T3 cells were used as a negative controls and the not specific cross-reaction of antibodies was excluded by incubating cells with isotype-matched immunoglobulins. pCX-hE5NT and pCX-hENTPD1 transfected cells were stained only with the corresponding antibody. pCX-hHO1 transfected cells were sorted and analyzed on the basis of EGFP expression.

### Immunofluorescence and Confocal Microscopy

Mock- and pCX-TRI-2A-transfected cells were seeded at 4x10^4^cells/well in 8-well chamber slides for 24 hours (LabTek Chamber slides, Thermo Fisher Scientific). The next day, cells were washed with PBS and fixed with methanol-acetone 1:1 for 10 min at -20°C. After fixation, cells were blocked with 1% BSA for 30 min. Fixed cells were incubated for 1 hour with primary antibodies, and for 30 minutes with the appropriate secondary antibodies diluted in 1% BSA (w/v) in PBS. Cells were then washed and counterstained with DAPI. The stained cells were mounted with mounting medium (Fluoromount; Sigma Aldrich) and analyzed by LSM 710 confocal microscope (Zeiss). Images were acquired by ZEN 2009 software (Zeiss).

### Immunoblotting

Control and transfected cell lines were lysed in RIPA buffer and whole protein concentration was quantified by Bradford assay (Sigma Aldrich). 20 μg of total protein extracts were separated in a 10% NuPAGE BT gel (Life Technologies) and then transferred onto nitrocellulose membranes using the iBlot system (Life Technologies). The membranes were probed with anti-hHO-1, anti-hE5NT, anti-hENTPD1 and anti-β-actin primary antibodies. For Nfkb nuclear translocation analyses, pCX-TRI-2A and control cells were exposed to TNF-α 50ng/ml alone or in combination with hemin 20μM and ATP 200μM for 16h and proteins were extracted with NE-PER Nuclear and Cytoplasmic Extraction Reagents kit (Thermo Scientific), following the manufacturer’s instructions. Protein concentration was determined by Micro BCA Protein Assay Kit (Thermo Scientific). 10 μg of nuclear protein extracts were separated in a 4–12% gradient NuPAGE BT gel (Life Technologies) and transferred onto nitrocellulose membranes. The membranes were probed with anti-Nf-kB1 p105/p50 and anti-Lamin B2 primary antibodies. Immunoreactive proteins were visualized by enhanced chemiluminescence (SuperSignal West Dura, Thermo Scientific) and digitally acquired using G:BOX (Syngene) instrument.

Densitometric analysis on Nf-kB/p50 and Lamin B2 bands was performed by using “Gel analyzer” function of ImageJ software[34].

### Heme Oxygenase activity assay

Heme oxygenase activity assay was performed as previously described [32,35]. Briefly, cells were lysed in lysis buffer (100 mM Tris-HCl, 150 mM NaCl, 1% Triton X-100, pH 7.4, supplemented with 2% protease inhibitor cocktail) and protein concentration was quantified by Bradford assay. 300 μg of crude lysate were with 15 μM hemin and 10U/ml recombinant biliverdin reductase A. The fluorescence of bilirubin was detected every 2 minutes in a fluorescence reader (Infinite M200; Tecan) at 37°C (excitation/emission wavelengths: 441/528nm). As a positive control of specific detection of heme oxygenase 1 activity, WT and mock transfected NIH3T3 cells were stimulated with 50 μM Cobalt-Protoporphyrin for 24 hours before the assay.

### E5NT and ENTPD1 activity assay

Ectonucleotidases activity assay was performed as previously described [36]. Briefly, NIH3T3 cells were washed with Hank balanced salt solution (HBSS) and pre-incubated for 15 minutes in HBSS supplemented with glucose (1mg/ml) and Adenosine Deaminase inhibitor, erythro-9-(2-Hydroxy-3-Nonyl) adenine, EHNA (5 μM). Cells were then incubated with 50 nmol/ml of AMP or ATP and supernatant samples were collected after 0, 5, 15, 30 minutes, frozen at -80°C and then analyzed by reverse phase HPLC [37] on Agilent 1100 HPLC instrument with a diode array detector.

### Cytotoxicity assay

WT and transfected cell lines were plated in quadruplicate in 96-well plate at 12x10^3^ cells per well. The day after plating, cells were incubated in culture medium with of without different combination of drugs (TNF-α 50 ng/ml, hemin 20 μM and ATP 200 μM) for 24h and 48h.

Cell toxicity was quantified by measurement of lactate dehydrogenase (LDH) release into the medium by using the LDH assay kit (Roche Diagnostics) following manufacturer’s instructions.

### Caspase activity assay

The Caspase-Glo 3/7 (Promega) assay was performed on WT and transfected cell lines grown in a white 96-well plate to reach 80% confluence, following manufacturer’s instructions. Briefly, after 16h and 24h of treatments with TNF-α 50 ng/ml, hemin 20 μM and ATP 200 μM, lyophilized Caspase-Glo 3/7 substrate was resuspended and added into each well. The contents of the wells were mixed gently and incubated at room temperature for 1 hour. Luminescent signal was measured with a 96 multi-well plate reader (Infinite M200; Tecan).

### Real time PCR analysis of TNF-α signaling genes

The expression of 84 TNF-α pathway-related genes in mouse were examined using the RT^2^ Profiler PCR array (PAMM-063C, SuperArray Bioscience). Control and transfected cell lines were treated with TNF-α 50ng/ml alone or in combination with hemin 20 μM and ATP 200 μM for 16h. Untreated cells for each cell line were used as a control. Total RNA was isolated from treated and control cells by using the RNeasy Mini kit (Qiagen) according to manufacturer’s instructions. RNA samples were treated with DNase to ensure elimination of genomic DNA, and the extracted RNA was converted to cDNA using the RT^2^ First Strand Kit from SuperArray Bioscience (Qiagen) following manufacturer’s protocol. PCR was performed with the RT^2^ Profiler PCR array system according to the manufacturer’s instructions using Step One Plus instrument (Applied Biosystems). The mRNA expression levels of each gene in each cell treatment were normalized using the expression of the housekeeping genes *B2m*, *Gapdh*, *Gusb*, *Hsp90ab1*, and *Actb*. The results were confirmed by quantitative reverse transcriptase-PCR performed using individual RNA samples from the cells in each group by Step One Plus instrument (Applied Biosystems). The primers used for real-time PCR are listed in S1 Table.

### Statistical Analysis

Statistical analyses were performed using SPSS v.19 for Mac and values of *p* ≤ 0.05 were considered to be statistically significant. LDH assay, caspase 3/7 assay and real-time PCR were independently performed 3 times. The results are represented as mean ± standard deviation (SD). Analysis of variance (one-way ANOVA) with Tukey *post hoc* test was used for multiple comparisons.

## Results

### Transgenic constructs design and generation of stable transfectants

**Fig 1:**
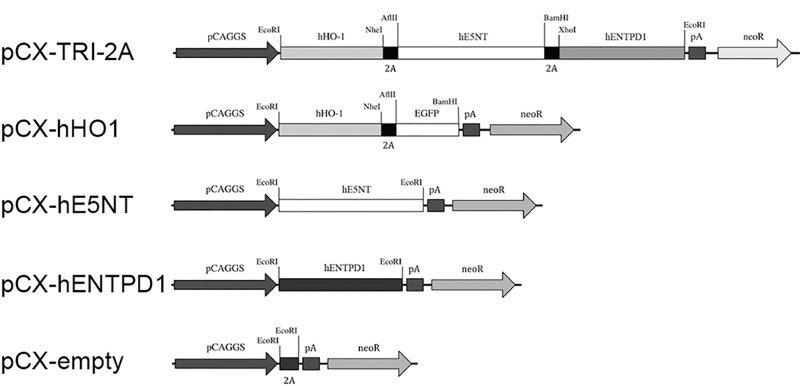
Schematic maps of tricistronic pCX-TRI-2A vector and all the constructs used in the work. The transgenic plasmid is composed of the CAGGS promoter, followed by the coding sequence of human HO-1 gene without the stop codon, fused in frame to the first F2A sequence (F2A), then the coding sequence of human E5NT gene without the stop codon, the second F2A sequence and the coding sequence of human ENTPD1 gene followed by a polyadenilation signal (pA).

We previously reported that the F2A technology can be used to link in frame three coding sequences obtaining a single open reading frame of 4.3 Kbp that can be expressed in eukaryotic cells as three discrete protein products [32]. In order to obtain a stable and unsilenced expression in eukaryotic cells we moved the sequence encoding for hHO1, hE5NT and hENTPD1 proteins under the control of the CAGGS promoter (Fig 1), to give the tricistronic pCX-TRI-2A plasmid. Single gene-expressing vectors have been produced as controls and cells transfected to investigate the contribution of each gene in the downregulation of the inflammatory response. hHO-1, hE5NT and hENTPD1 coding sequences were cloned into the same vector backbone used to produce pCX-TRI-2A plasmid (Fig 1).

**Fig 2:**
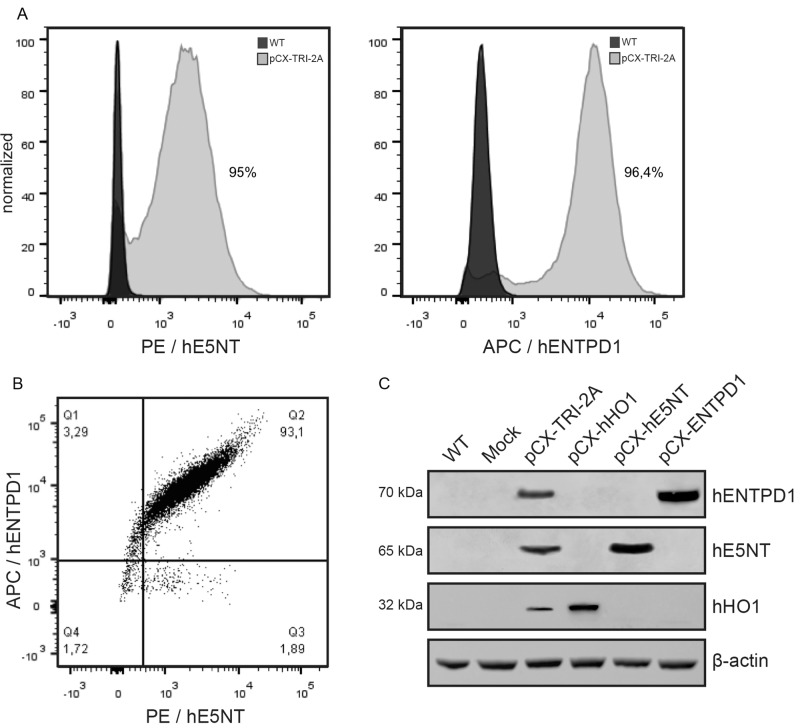
pCX TRI 2A-transfected cells were enriched via FACS for the expression of hE5NT and hENTPD1. Expression analysis on sorted transfectants showed that E5NT and ENTPD1 were expressed by more than 95% of the pCX-TRI-2A-transfected cells (**A**) and that more than 93% of those cells expressed both the two ecto-enzyme simultaneously (**B**). (**C**) The three human proteins were found strongly expressed into the transfected cells after sorting, and expression levels were unaffected by the number of genes in the construct, as there was no evidence of incomplete separation of individual proteins.

Murine NIH3T3 cells were electroporated with pCX-TRI-2A and control plasmids, and selected for neomycin resistance for one week. In order to verify the presence and the functionality of pCX-TRI-2A vector, genomic DNA and total RNA were extracted from transfected cells and analyzed for the presence of the exogenous molecules. PCR analysis on genomic DNA using transgene-specific oligonucleotides confirmed the genetic modification of the cells (S1 Fig). RT-PCR analyses on total RNA, using oligonucleotides specific for transgenic transcript, also confirmed the correct transcription of the tricistronic cassette (S2 Fig). In order to enrich the population of transfected cells, the expression of hENTPD1, hE5NT or EGFP was firstly assessed by flow cytometry (data not shown), and then those cells expressing the human proteins were sorted and expanded. pCX-TRI-2A transfected cells were FACS-sorted on the basis of high hE5NT and hENTPD1 expression. Sorted cells were expanded in culture for ten days and then analyzed for the expression of both hENTPD1 and hE5NT: approximately 93% of cells were expressing both human proteins (Fig 2A and 2B). pCX-hHO1, pCX-hE5NT and pCX-hENTPD1 transfected cells were sorted and analyzed for EGFP, hE5NT and hENTPD1 expression respectively. After sorting >98% of cells expressed the exogenous protein, conversely no signal was detected in WT and mock-trasnfected cells (S3 Fig).

To verify if the enriched cells overexpressed all the three human proteins, controls and transfected cell lysates were analyzed by immunoblotting. As expected, all the three human proteins were found to be strongly expressed in pCX-TRI-2A cells and to have the correct molecular weight. Single gene transfected cell groups showed in each case higher expression levels (Fig 2C) as compared to pCX-TRI-2A cells.

### The tricistronic transgene encodes for all the three human proteins with a correct subcellular localization

**Fig 3:**
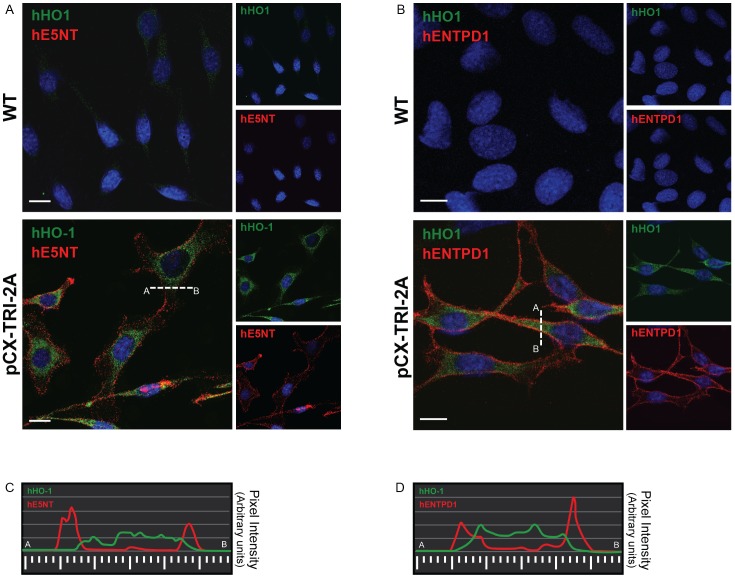
All the three exogenous proteins were correctly localized in pCX TRI 2A-transfected cells. WT and pCX-TRI-2A-transfected cells were co-stained with anti-hHO1 and anti-hE5NT antibodies (**A**) or with anti-hHO1and anti-hENTPD1antibodies (**B**). Transfected cells positive to hE5NT or hENTPD1 (red) were also positive to hHO1 (green). hE5NT and hENTPD1 localized on the cell surface, while hHO1 had a perinuclear and/or ER membranes cytoplasmic localization. (**C**-**D**) Plot of the signal intensity for hE5NT and hENTPD1 (red) and hHO1 (green) along the line drawn in A and B indicated that the most intense hE5NT and hENTPD1 signals are not colocalized with hHO1 signal.

Since it has been demonstrated that different subcellular localization might influence the expression pattern of target genes coupled to the 2A peptide [38], we investigated if the human proteins encoded by the muticistronic 2A-based transgene had a correct subcellular localization by immunofluorescence and confocal analysis. Co-staining analysis for hE5NT and hHO1 or for hENTPD1 and hHO1 indicated that in pCX-TRI-2A transfected cells both the hENTPD1 and hE5NT signals had, as expected, a distribution pattern similar to that of plasma membrane proteins, whereas hHO1 signal was detected mainly in the perinuclear area suggesting, for this protein, a cytoplasmic localization related to the ER (Fig 3A and 3B). Pixel intensity analysis further confirmed the correct localization of the exogenous proteins (Fig 3C and 3D)

### pCX-TRI-2A mediates the increase of HO-1 and ENTPD1/E5NT activity

Since it has been reported that the function of the upstream protein in 2A-based constructs could be disrupted by the residual 2A peptide fused to its C-terminus [6], we carefully investigated the functionality of all the three exogenous proteins.

**Fig 4:**
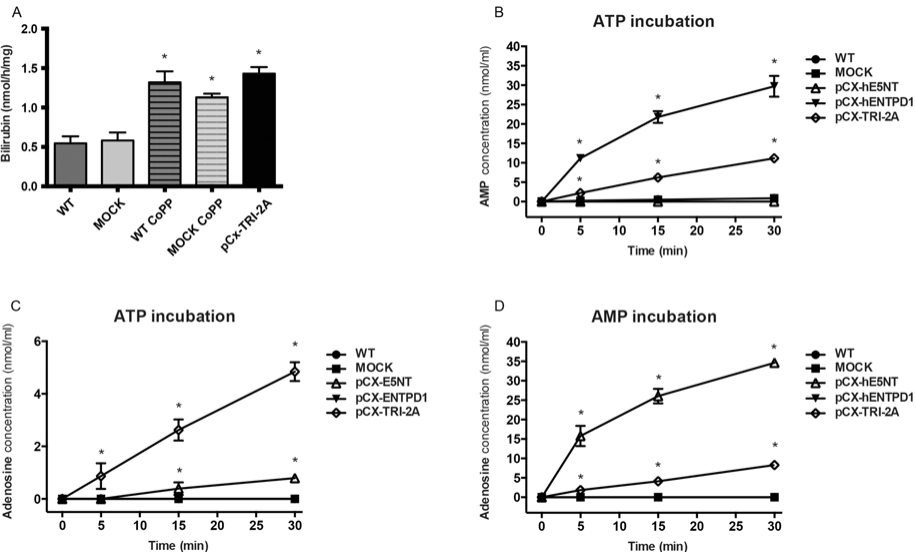
Heme oxygenase-1 and ectonucleotidases functional assays. (**A**) Heme Oxygenase 1 activity assay on NIH3T3 cells. Lysates from WT, mock- and pCX-TRI-2A-transfected cells were incubated for 2 hours with Hemin, BSA, Biliverdin Reductase A in reaction buffer as described in Materials and Methods. As positive control of the assay, wt or mock-transfected cells were pre-stimulated with 50 μM of HO1 inducer, Cobalt Protoporphyrin for 24 hours (wt CoPP, mock CoPP). Enzymatic activity is reported as nanomoles of bilirubin per hours per milligrams of protein extract. Data are expressed as mean ± SEM of 3–4 independent experiments. *p<0.05 versus wt and mock-transfected cells. (B) ENTPD1-mediated AMP production and (C) E5NT-mediated adenosine production by wild type, mock and pCX-TRI-2A transfected-cells. Cells were incubated with 50 μM ATP for 30 min. The nucleotide content of supernatants collected at 0, 5, 15, 30 min time points was measured by reverse phase-HPLC as detailed in Material and Methods. Data shown are mean ± S.D. (n = 3). *p<0.05 versus all groups. (D) E5NT-mediated adenosine production by wild type, mock and transfected-cells. Cells were incubated with 50 μM AMP for 30 min. The nucleotide content of supernatants collected at 0, 5, 15, 30 min time points was measured by reverse phase-HPLC ad detailed in Material and Methods. Data shown are mean ± S.D. (n = 3). *p<0.05 versus all groups.

The enzymatic activity of HO-1 in pCX-TRI-2A-transfected cells was evaluated by measuring the fluorescence of bilirubin during the incubation of lysates from WT, mock and pCX-TRI-2A-transfected cells with hemin. As shown in Fig 4A, the HO-1 activity in pCX-TRI-2A-transfected cells was about 2.5 fold higher than the basal activity seen in controls (1.43 ± 0.08 nmol/h/mg versus 0.54 ± 0.08 and 0.58 ± 0.1 nmol/h/mg respectively in WT and mock transfected-cells, p<0.05). Moreover, no significant differences were observed between pCX-TRI-2A and control cell lines (WT and mock-transfected cells) previously treated with CoPP. These data showed that the exogenous expression of hHO-1, encoded by pCX-TRI-2A plasmid, was comparable to the CoPP induced expression levels of endogenous HO-1.

The enzymatic activity of the ENTPD1/E5NT system encoded by the tricistronic plasmid was evaluated by an extracellular nucleotide metabolism assay. Confluent control and transfected cells were incubated with ATP or AMP and the nucleotide content of supernatant samples collected at 0, 5, 15 and 30 min was analysed by HPLC [32,36].

As shown in Fig 4B, in pCX-TRI-2A-transfected cells the supernatant content of AMP, which is the product of ENTPD1 enzymatic activity, was significantly higher than in controls (WT and mock-transfected cells) and pCX-hE5NT-transfected cells at every time points (*p*<0.05). As expected, the production of AMP in the supernatant from the pCX-hENTPD1-transfected cells was even higher at every time point (*p*<0.05 versus all the other experimental groups).

On the other hand, a significant increase of adenosine concentration has been detected in medium from pCX-TRI-2A-transfected cells (4.8 ± 0.3 μM after 30 min, Fig 4C, *p*<0.05 vs. all the other experimental groups). A slight increase has been observed in pCX-hE5NT-transfected cells (0.7 ± 0.05 μM after 30 min, Fig 4C, *p*<0.05) while no adenosine formation has been detected in WT, mock and pCX-hENTPD1-transfected cells, suggesting a very low AMPasic activity in these latter cell groups (Fig 4C). As control of hE5NT activity, confluent cells were incubated with 50 μM AMP for 30 min. As shown in Fig 4D, formation of adenosine in pCX-hE5NT-transfected cells increased significantly to 34.6 ± 0.1 μM. A significant increase of adenosine production has been observed also in pCX-TRI-2A-transfected cells (8.2 ± 0.3 μM) as compared to WT, mock and pCX-hENTPD1-transfected cells in which no detectable levels have been observed in all the time of incubation (Fig 4D).

These data suggested that the overexpression of both ectonucleotidases is efficient in the removal of the pro-inflammatory ATP and AMP molecules and, at the same time, to increase the production of the anti-inflammatory adenosine.

In conclusion, these data suggested that the simultaneous expression of the three genes does not alter the enzymatic activity of each of the pCX-TRI-2A encoded genes and that they were able to mediate the production of anti-inflammatory molecules in the pCX-TRI-2A transfected cells.

### The expression of hHO-1, hE5NT and hENTPD1 protects cells from TNF-α-mediated cytotoxicity and apoptosis

We next evaluated whether the simultaneous expression and activity of this new combination of human proteins confers protection against inflammatory stimuli and if this protection would be higher than the protective effect given by each single human protein. To this extent, controls and transfected cells were exposed to 50 ng/ml of TNF-α alone or in combination with appropriate molecules that served as a substrate for the enzymatic activity of exogenous proteins, hemin (20 μM) and/or ATP (200 μM), for up to 48 hours and the cytotoxicity was measured by LDH assay.

**Fig 5:**
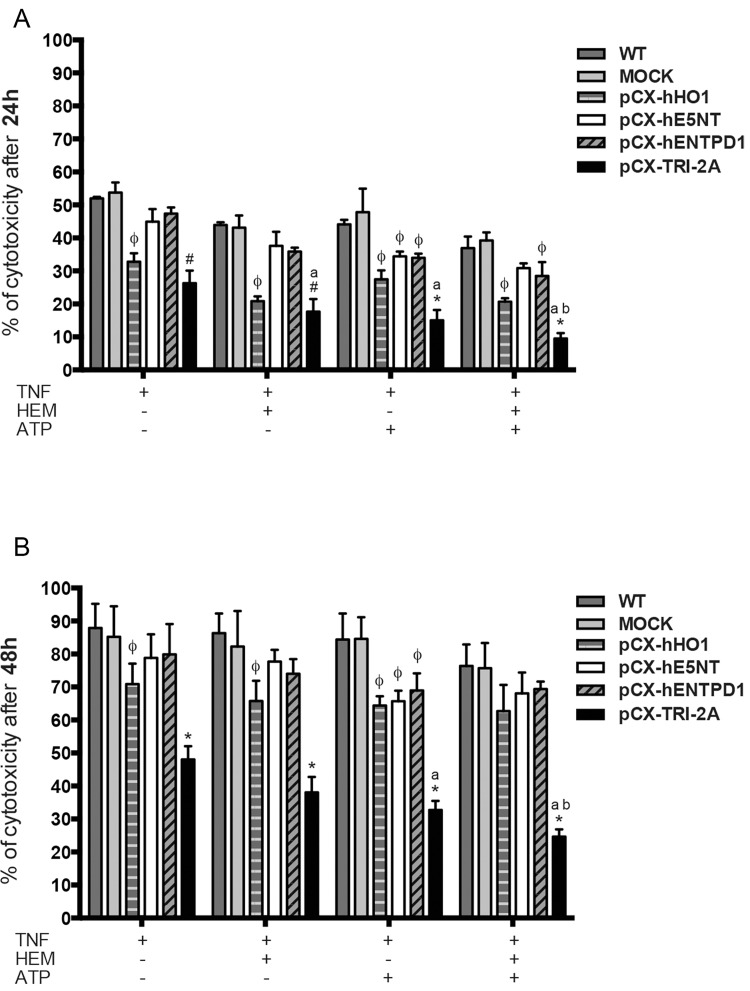
pCX-TRI-2A transfected cells are protected against TNF-α-induced cytotoxicity. WT cells and transfected cell lines were incubated with 50 ng/ml TNF-α for 24 h (**A**) and 48 h (**B**) alone or in combination with 20 μM hemin and/or 200 μM ATP. Cytotoxicity was assessed by lactate dehydrogenase (LDH) release and expressed as follows: relative cytotoxicity (%)  =  [(*A* e − *A* c) / (*A* b − *A* c)] × 100 (%), where ‘*A* e’ is the experimental absorbance, ‘*A* b’ is the absorbance of lysed controls and ‘*A* c’ is the absorbance of untreated controls. The data are expressed as mean ± SD of three independent experiments. [#] indicates significant difference between pCX-TRI-2A and all the other groups, except for pCX-hHO1, within the same treatment (ANOVA, *p*<0.05). [*] indicates significant difference between pCX-TRI-2A and all the other groups within the same treatment (ANOVA, *p*<0.05); [ϕ] indicates a significant difference between single gene-transfected cells and WT/mock within the same treatment (ANOVA, *p*<0.05); [a] indicates significant difference as compared to TNF-α treatment alone within the same cell type (ANOVA, *p*<0.05); [b] indicates a significant difference as compared to TNF-α + hemin treatment within the same cell type (ANOVA, *p*<0.05).

As shown in Fig 5, the percentage of dead cells in pCX-TRI-2A transfected cells was 26.2 ± 3.8% after 24 hours (Fig 5A) and 48 ± 3.9% after 48 hours (Fig 5B) of treatment with TNF-α alone, which was significantly lower as compared to all other cell lines at 48 hours of treatment (*p*<0.05), even in absence of enzymatic substrate of human genes, and to all the other cell lines (*p*<0.05) except to pCX-hHO1 transfected cells at 24 hours of treatment. On the other hand, the administration of enzymatic substrates, hemin, ATP or both, to pCX-TRI-2A transfected cells treated with TNF-α, induced a further reduction of cytotoxicity (17.6 ± 3.8%, 15 ± 3.1% and 9.5 ± 1.6% respectively) as compared to the same cells treated with TNF-α alone at 24 hours (26.2 ± 3.8%, *p*<0.05). Similarly, the addition of both hemin and ATP to TNF-α treatment induced a 24.6 ± 2.2% of cell death in pCX-TRI-2A cells at 48 hours (Fig 4B), which was significantly lower than cytotoxicity of same cells treated with TNF-α alone (48 ± 3.9%, *p*<0.05).

**Fig 6:**
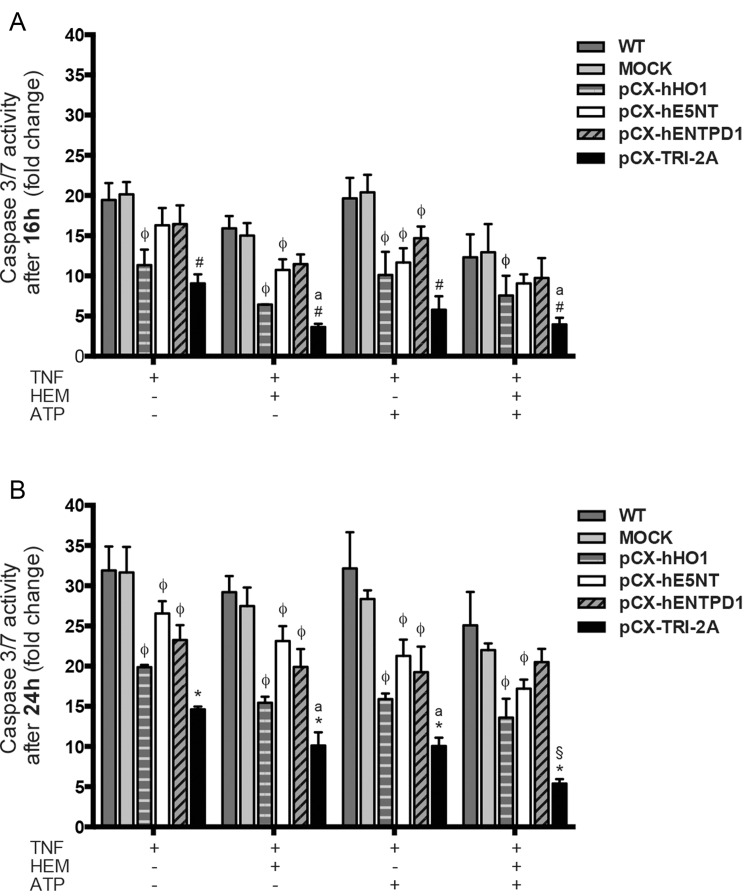
pCX-TRI-2A transfected-cells are protected against TNF-α-induced apoptosis. Caspase 3/7 activities were determined in all cell groups after 16h (**A**) or 24h (**B**) of incubation with 50 ng/ml TNF-α alone or in combination with 20 μM hemin and/or 200 μM ATP. Expression of the three human genes in TG cells significantly reduced the activation of effector caspases 3/7. The data are expressed as mean ± SD of three independent experiments. [#] indicates significant difference between pCX-TRI-2A and all the other groups, except for pCX-hHO1, within the same treatment (ANOVA, *p*<0.05). [*] indicates significant difference between pCX-TRI-2A and all the other groups within the same treatment (ANOVA, *p*<0.05); [ϕ] indicates a significant difference between single gene-transfected cells and WT/mock within the same treatment (ANOVA, *p*<0.05); [a] indicates significant difference as compared to TNF-α treatment alone within the same cell type (ANOVA, *p*<0.05); [§] indicates a significant difference as compared to all the other treatments within the same cell type (ANOVA, *p*<0.05).

Since soluble TNF-α can lead to cell apoptosis [39,40], and considering that the activity of the three human proteins has been reported to exert anti-apoptotic effects [41,42], we investigated their potential role in protection against cell death induced by TNF-α via a caspase 3/7 activity assay. As shown in Fig 6, pCX-TRI-2A-transfected cells were protected against apoptotic death after both 16 (Fig 6A) and 24 (Fig 6B) hours of TNF-α challenging as compared to WT and mock cells (*p<*0,05). Furthermore, pCX-TRI-2A-transfected cells resulted better protected from TNF-α-induced caspase activation as compared to pCX-hE5NT, pCX-hENTPD1 and pCX-HO1 after 24 hours of treatment (*p*<0.05). On the other hand, the better protection was also observed at 16 hours of treatment as compared to pCX-hENTPD1 (*p*<0.05) and pCX-hE5NT (*p*<0.05) but not as compared to pCX-HO1. The addition of hemin (20 μM) to pCX-TRI-2A-transfected cells treated with TNF-α further reduced caspase activation after 16 hours (Fig 6A) and this anti-apoptotic effect was still observed at 24 hours (Fig 6B) as compared to pCX-TRI-2A-transfected cells treated with TNF-α alone. This reduced TNF-α-dependent caspase activation was observed also in presence of ATP (200 μM) to pCX-TRI-2A transfected cells after 24 hours of TNF-α treatment as compared to pCX-TRI-2A-transfected cells treated with TNF-α alone (Fig 6B). The combined treatment with TNF-α, hemin and ATP inhibited apoptosis at 16 hours in pCX-TRI-2A transfected cells (3.97 ± 0.81; fold change as compared to TNF-α treatment alone, *p* <0,05), similarly to the observed anti-apoptotic effect of hemin (Fig 6A). Interestingly, in pCX-TRI-2A transfected cells treated with TNF-α together with both hemin and ATP after 24 hours, it was observed an anti-apoptotic effect significantly greater than in all the other treatment groups (*p* <0,05, Fig 6B).

To further evaluate the protective response to the TNF-α injury after a persistent exposition to the pro-inflammatory cytokine, a propidium iodide incorporation assay was performed. Consistently with the results of cytotoxicity and caspase assays, the pCX-TRI-2A-transfected cells were protected against TNF-α injury as compared to WT cells up to 72 hours (S4 Fig).

Taken together, these data suggest that the expression of the three genes is protective against TNF-α-induced cytotoxicity and apoptosis and that the protection is more effective when all the three genes, hHO1, hENTPD1 and hE5NT are simultaneously present and exerting their enzymatic activity.

### Molecular characterization of anti-inflammatory response mediated by the combination of the human genes

**Fig 7:**
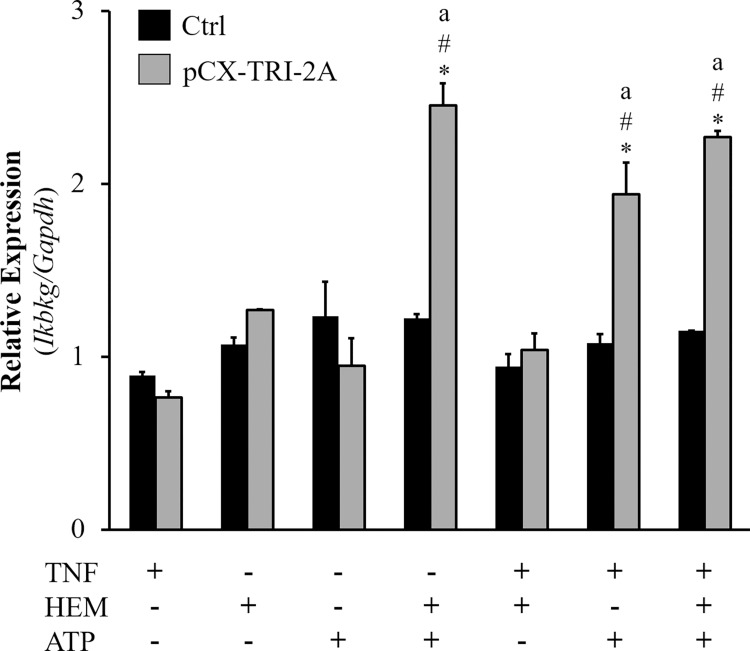
Changes in *Ikgkb* (*Nemo*) mRNA expression in control (Ctrl) and pCX-TRI-2A-transfected cells. Cells were incubated with 50 ng/ml TNF-α for 16h, alone or in combination with 20 μM hemin and/or 200 μM ATP. Murine *Ikgkb* mRNA was quantified by real-time PCR. The data (mean±SD of three independent experiments), normalized for *Gapdh* gene, are expressed as fold change respect to the untreated cells. [*] indicates a significant difference between pCX-TRI-2A-transfected cells and Ctrl cells within the same treatment (t Student, *p*<0.05); [#] indicates a significant difference as compared to untreated cells within the same cell type (ANOVA, *p*<0.05) [a] indicates a significant difference as compared to TNF-α treatment alone within the same cell type (ANOVA, *p*<0.05).

Trying to unravel the molecular mechanism of the anti-inflammatory response mediated by the combination of the three human genes in pCX-TRI-2A transfected cells, the expression of 84 TNF-α pathway-related genes was analyzed by quantitative RT^2^–PCR Profiler Array (Qiagen). By this screening several genes emerged to be differentially modulated in control and pCX-TRI-2A-transfected cells by comparing each treatment with the respective untreated cells (S5 and S6 Figs, S2 Table). Among these, *Ikbkg (Nemo)*, a gene encoding for the regulating subunit of IKK complex which is involved in the Nf-kB activation in response to cytokine exposure, was selected for further validation because it has been observed to have an unexpected transcriptional up-regulation in pCX-TRI-2A-transfected cells. A significant increase of *Ikbkg* expression was observed only in pCX-TRI-2A-transfected cells treated with ATP plus TNF-α or Hemin or both (Fig 7), as compared to the respective untreated cells (*p*<0.05) or to TNF-α treated cells (*p*<0.05). These data suggest that pCX-TRI-2A-transfected cells exposed to TNF-α injury up-regulate the *Ikbkg* gene.

**Fig 8:**
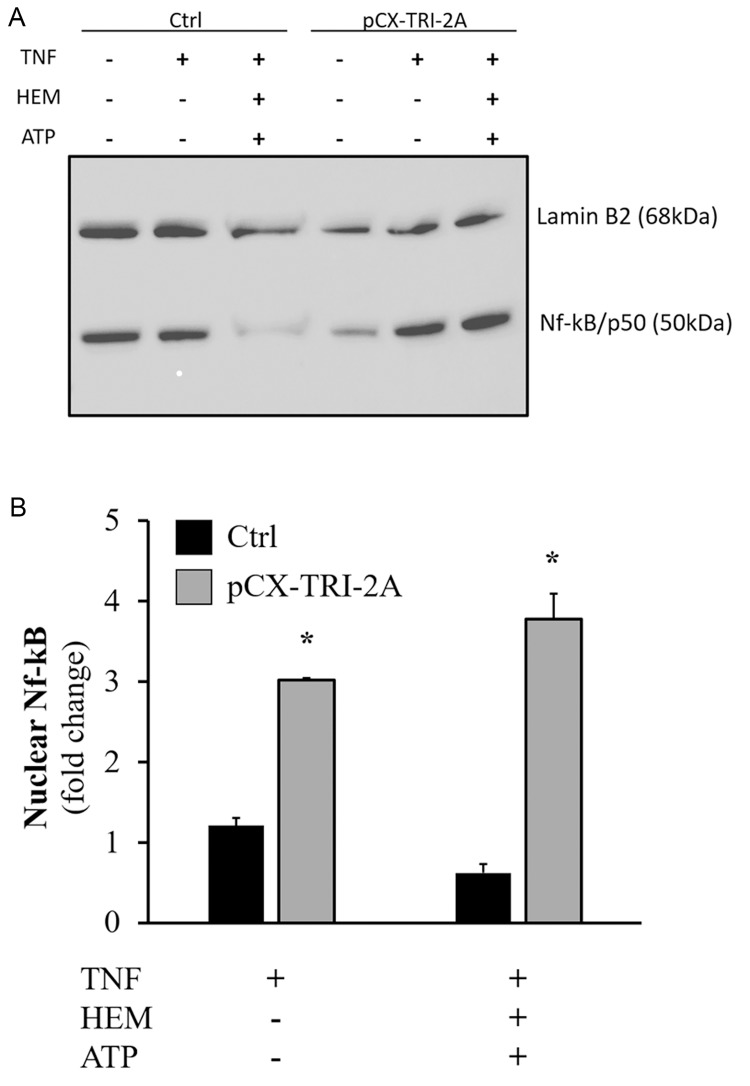
Nf-kB1 nuclear translocation in control (Ctrl) and pCX-TRI-2A-transfected cells. Cells were incubated with 50 ng/ml TNF-α for 16h, alone or in combination with 20 μM hemin and 200 μM ATP. Murine Nf-kB1/p50 protein was detected in nuclear extracts of Ctrl and pCX-TRI-2A-transfected cells by immunoblotting analysis (**A**). Densitometric analysis for Nf-kB1/p50, normalized for the Lamin B2 signal, showed a 60% or 83.6% increase in Nf-kB1/p50 level in pCX-TRI-2A-transfected cells treated with TNF-α alone or in combination with hemin and ATP, respectively, as compared to control cells (**B**). Densitometric analysis was performed by using “Gel analyzer” function of ImageJ software. Data are shown as mean ± S.D from at least three independent experiments. [*] indicates a significant difference between pCX-TRI-2A-transfected cells and control cells within the same treatment (t Student, *p*<0.05).

Since it is known that, after TNF-α exposure, the cells respond with an Nf-kB activation leading to a pro-survival pathway, and that this response is strictly dependent from IKK complex[39], we investigated if the nuclear translocation of Nf-kB was correlated with the *Nemo* expression profiles in pCX-TRI-2A-transfected and control cells in presence of TNF-α alone or in combination with hemin and ATP. To this extent, immunoblot and densitometric analyses were performed to evaluate the presence of Nf-kB/p50 active form into the nucleus of control and pCX-TRI-2A-transfected cells after 16h of treatment with TNF-α alone or in combination with hemin and ATP (Fig 8). The levels of nuclear Nf-kB/p50 in pCX-TRI-2A-transfected cells were 3,02-fold higher after treatment with TNF-α alone and 3,77-fold higher after treatment with TNF-α in combination with hemin and ATP as compared to untreated pCX-TRI-2A-transfected cells (*p*<0.05, Fig 8B), while no significant differences were observed in control cells among treatments (Fig 8B). Additionally, levels of nuclear Nf-kB/p50 in pCX-TRI-2A transfected cells were notably increased as compared to control cells within the same treatment (*p*<0.05, Fig 8B).

**Fig 9:**
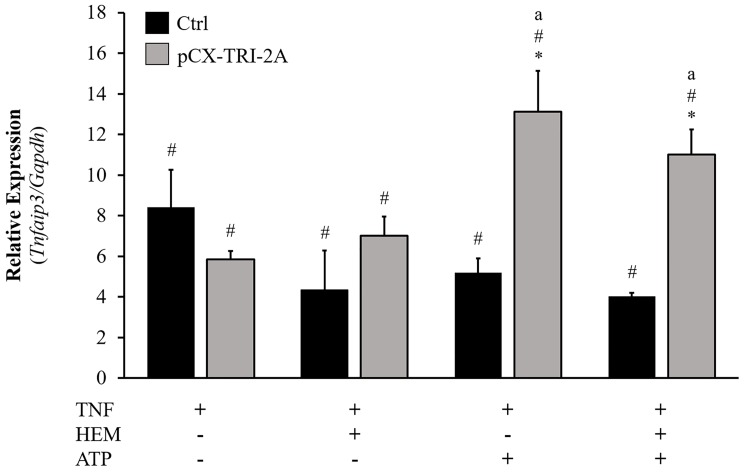
Changes in *Tnfaip3* (*A20*) mRNA expression in control (Ctrl) and pCX-TRI-2A-transfected cells. Cells were incubated with 50 ng/ml TNF-α for 16h, alone or in combination with 20 μM hemin and/or 200 μM ATP. Murine *Tnfaip3/A20* mRNA was quantified by real-time PCR. Data (mean±SD of three independent experiments) are normalized for *Gapdh* gene and expressed as fold change respect to the untreated cells. [*] indicates a significant difference between pCX-TRI-2A-transfected cells and control cells within the same treatment (t Student, *p*<0.05); [#] indicates a significant difference as compared to untreated cells within the same cell type (ANOVA, *p*<0.05); [a] indicates a significant difference as compared to TNF-α treatment alone within the same cell type (ANOVA, *p*<0.05).

In order to further investigate the possible correlation between the expression of *Nemo* and the Nf-kB activity, we came back to the RT^2^ array results and selected the *Tnfaip3/A20* gene, one of the Nf-kB transcriptional targets and coding for a well-known anti-apoptotic protein [43,44], for further validation. The expression of *Tnfaip3* gene was then analysed in control and pCX-TRI-2A-transfected cells after 16h of treatments. As shown in Fig 9, the up-regulation of *Tnfaip3* was observed, as expected, in both control and pCX-TRI-2A-transfected cells treated with TNF-α, alone or in combination with hemin and/or ATP (*p*<0.05, as compared to untreated cells). Moreover, pCX-TRI-2A-transfected cells showed a further increase in *Tnfaip3* induction after TNF-α treatment in combination with ATP and with both ATP and hemin (*p*<0.05, as compared to TNF-α treated cells), that it was not observed in control cells (Fig 9).

Taken together, these data suggested that in pCX-TRI-2A-transfected cells exposed to TNF-α in combination with the exogenous protein substrates Nf-kB translocated into the nucleus and function as transcription factor for the expression of the pro-survival gene *Tnfaip3*.

## Discussion

The results shown in the present paper contribute to the understanding of the protective role of a novel combination of human genes, HO-1, ENTPD1 and E5NT, against inflammatory stimuli such as TNF-α. Each human gene used in this study has been reported to have anti-inflammatory and anti-apoptotic properties when overexpressed or induced in the cells or organisms [21,41,45–48]. HO-1 was shown in various experimental models of xenograft rejection to be highly expressed in the endothelium and smooth muscle cells of accommodated heart and lung xenografts, suggesting its critical role in preventing the development of chronic graft dysfunction in a clinical setting [49–51]. Human ENTPD1 overexpression protected transgenic mouse cardiac xenografts from antibody-mediated rejection [52] as well as pancreatic islets from “Instant Blood Mediated Inflammatory Reaction” (IBMIR) after exposure to human blood [53]. These evidences suggest that ENTPD1 could address the coagulation and thrombotic disorders that still limit the graft survival [54]. In addition, the overexpression of human E5NT has been demonstrated to exert beneficial effects in porcine endothelial cells with increased adenosine production and protection against human NK cells mediated cytotoxicity [24]. For the first time we report here the protective effects given by the simultaneous expression and enzymatic activities of the combination of these genes as compared to their use as a single agent in cells exposed to inflammatory stimuli.

The coding sequences of the three human genes were included in an expression cassette that allowed the simultaneous translation of three proteins starting from a single mRNA by using the F2A technology [4,55]. The western blotting analyses, performed on protein extracts of pCX-TRI-2A and control plasmids transfected cells, confirmed the enrichment of cells expressing exogenous proteins obtained by FACS and no evidence of incomplete separation of individual proteins was found. The cells transfected with plasmids expressing each of the three genes alone were found to have higher levels of exogenous protein as compared to the pCX-TRI-2A transfected cells, and this is probably due to the smaller size of the single gene expressing plasmids as compared to the pCX-TRI-2A plasmid as previously reported [56]. The order of genes encoded by the expression cassette was designed to maximize the likelihood of the correct processing and maturation of each protein product [32] and we found the expected subcellular localization for hHO1, hE5NT and hENTPD1 in pCX-TRI-2A transfected cells.

Taken together, the expression analyses data confirmed that the application of the F2A system and the design of the multi-cistronic construct allowed the expression of three exogenous proteins that were correctly processed and localized within the cells.

Next, to verify the absence of possible interference on protein function in F2A-based encoded peptides [6], we focused on the investigation of enzymatic activity of each gene encoded by the multi-cistronic plasmid. The activity of HO-1, evaluated by means of measuring bilirubin’s fluorescence during the incubation with hemin [57], was found to be higher in pCX-TRI-2A transfected cells as compared to mock-transfected or untransfected cells (Fig 4A). Furthermore, the HO-1 activity of pCX-TRI-2A transfected cells was similar to the activity observed in control cells previously treated with a known HO-1 inducer, CoPP, suggesting that the pCX-TRI-2A transfected cells constitutively express HO-1 at amounts comparable to those induced in stress conditions within the cells. The enzymatic activities of ENTPD1 and E5NT was measured by an extracellular nucleotide metabolism assay [32,37]. The production of AMP following incubation with ATP was significantly higher in pCX-hENTPD1 and pCX-TRI-2A transfected cells as compared to all the other experimental groups, which is consistent with the overexpression of ENTPD1 protein only in these two cell types. The pCX-hENTPD1 transfected cells had a significantly higher content in AMP as compared to pCX-TRI-2A transfected cells, suggesting that in the latter the AMP is produced in less amount or enzymatically converted in adenosine. In fact, the analysis of adenosine production after incubation with ATP revealed a significantly higher production in pCX-TRI-2A transfected cells as compared to all the other cell types, suggesting that, only in pCX-TRI-2A cells, the ATP could be converted in ADP and AMP by ENTPD1 and AMP converted in adenosine by E5NT. In order to evaluate the specific enzymatic activity of E5NT, the cells were incubated with AMP and adenosine production in the supernatant was measured. Only in the supernatant of hE5NT expressing cells (pCX-hE5NT and pCX-TRI-2A transfected cells) adenosine was detected, with significantly higher levels in pCX-hE5NT as compared to pCX-TRI-2A transfected cells (Fig 4D), and this finding is consistent with the higher amount of E5NT protein detected in pCX-hE5NT transfected cells (Fig 3B). The enzymatic assays demonstrated that the three proteins encoded by the multi-cistronic cassette were fully functional and allowed the increased production of their enzymatic products.

In order to evaluate the protective effects of the combination of human genes in the cells against inflammatory stimuli, pCX-TRI-2A and control cells were exposed to TNF-α alone or in combination with appropriate molecules that served as a substrate for the enzymatic activity of exogenous proteins and cytotoxicity and caspase assays were performed. TNF-α was chosen to mimic an inflammatory settings because it plays one of the most important roles in inflammation and in inflammatory conditions [26,58,59]. The expression of the combination of the three genes, better than the expression of each single gene, protected the cells against TNF-α induced cytotoxicity even in absence of enzymatic substrate of human genes. The expression of single genes appeared to be somehow protective against TNF-α-induced cytotoxicity as compared to WT or mock-transfected cells only if appropriate enzymatic substrate was administered to cells and the protective effect was observed exclusively when TNF-α-treated cells were treated with only one substrate (hemin or ATP). Taking into account that caspases play an important role in TNF-α-induced apoptotic cell death [39], we determined caspase activity in pCX-TRI-2A transfected and control cell types at 16 and 24 hours post-incubation with inflammatory stimuli (TNF-α) alone or in combination with enzymatic substrates of human genes (hemin and/or ATP). After 16 hours of treatment, cells overexpressing HO-1 (pCX-hHO1 and pCX-TRI-2A transfected cells) were protected from TNF-α-induced apoptosis, suggesting that the anti-apoptotic effect was mediated mainly by HO-1. On the other hands, the expression of the combination of the genes was better protective, as compared to the expression of each single gene, at 24 hours of TNF-α treatment and it was more effective in presence of enzymatic substrates of the three human proteins. It is well known that HO-1 is able to prevent cells death via inhibition of caspase-3 [13], and by suppression of TNF-α/TNFR1-mediated apoptotic signaling [41]. In presence of its enzymatic substrate, hemin, HO1 further confers cell protection by producing CO, which in low dose mediates anti-apoptotic and anti-inflammatory effects [60–62], and biliverdin, which is further converted by biliverdin reductase to bilirubin, a potent anti-oxidant and anti-inflammatory agent [63–65]. Exogenous administration of hemin has been shown to exert beneficial effects in models of hepatic ischemia-reperfusion injury [66] and to prolongs cardiac xenograft survival [67]. On the other hands, extracellular ATP is converted to adenosine by the combined activities of ENTPD1 and E5NT [21]. Porcine endothelial cells were protected against TNF alpha mediated apoptosis by adenosine signaling through A2A [68]. Adenosine signaling through A2B has been shown to confer protection against cardiac ischemic damages by activating salvage kinase pathway members, Akt, ERK 1/2 and GSK-3beta [25,69].

Taken together, these findings suggest that the simultaneous presence and activity of the three genes is necessary to further improve a persistent protection against TNF-α injury as compared to the effects induced by the expression of each single gene.

In order to better understand how the combined activity of the two systems represented by hHO-1 and hENTPD1/hE5NT were able to protect pCX-TRI-2A transfected cells against TNF-α-mediated injury we investigated the molecular mechanisms involved in TNF-α pathway. To this extent, TNF-α pathway-related genes were analysed by RT^2^ array and, among the several genes resulted to be differentially modulated between control and pCX-TRI-2A transfected cells, we initially focused our attention on *Ikbkg* (*Nemo*). It has been demonstrated that, upon TNF-α binding, the TNFR1 forms two different and consecutive complexes. The complex I controls the expression of anti-apoptotic proteins and the complex II triggers cell death process [26,39,40,70]. The complex I is responsible for the downstream activation of the transcriptional activation of NF-kB through the regulatory subunit of the IKK complex, Nemo [39], which is known to act at protein level [71] and it is not expected to be transcriptionally regulated. In this context, Nf-kb promotes pro-survival signaling within the cells. The gene expression analysis of *Nemo* showed a significant up-regulation of this gene in pCX-TRI-2A transfected cells treated with ATP plus TNF-α or Hemin or both at 16 hours post-treatment (Fig 7), as compared to untreated or to TNF-α treated pCX-TRI-2A-transfected cells. This suggested that *Nemo* modulation could be dependent to ATP administration to cells. Moreover, expression of *Nemo* was markedly lower in control cells compared to pCX-TRI-2A-transfected cells within these three treatments (Fig 7). On the other hand, TNF-α plus hemin administration to pCX-TRI-2A-transfected cells did not induce *Nemo* expression. This behaviour of the *Nemo* regulation in presence of hemin needs further experiments to be explained, although it can be hypothesized a protective effect of the HO-1 activity that could have abrogated the cell’s need of *Nemo*’s up-regulation.

Since it is known that, after TNF-α exposure, the regulatory subunit of IKK complex, Nemo, is responsible for Nf-kB activation and translocation into the nucleus [39], we analysed the nuclear p50 accumulation in pCX-TRI-2A-transfected and control cells exposed to TNF-α alone or in combination with hemin and ATP in order to verify if the up-regulation of Nemo was correlated with Nf-kB mediated pro-survival effect. Nuclear translocation of Nf-kB was significantly higher in pCX-TRI-2A-transfected cells as compared to control cells (Fig 8) and this finding was consistent with the observed increased resistance to cytotoxicity (Fig 5) and apoptotic cell death (Fig 6) as compared to control cells. Nevertheless, the Nf-kB activation was observed both in pCX-TRI-2A-transfected cells treated with TNF-α alone and with TNF-α plus hemin and ATP (Fig 8), suggesting that the pro-survival activation of Nf-kB could be due to other factors besides Nemo in cells treated with TNF-α alone. To verify this hypothesis, we came back to the RT^2^ array analysis and selected the *Tnfaip3*/*A20* gene, encoding for a zinc finger protein known as a Nf-kB-induced negative feedback regulator and inhibitor of apoptosis [43,44]. The expression profile of this gene revealed that, as expected, both control and pCX-TRI-2A-transfected cells adequately responded to TNF-α (alone) exposure by inducing the expression of *Tnfaip3* gene (Fig 9). On the other hand, pCX-TRI-2A-transfected cells significantly up-regulated the expression of the anti-apoptotic *Tnfaip3* gene, as compared to control cells, when treated with TNF-α plus ATP or with TNF-α plus ATP and hemin (Fig 9). This finding was, again, consistent with the cell death resistance of p-CX-TRI-2A-transfected cells treated with TNF-α plus ATP and hemin (Figs 5 and 6). Noteworthy, extracellular ATP is a potent pro-inflammatory molecule and we speculate that pCX-TRI-2A-transfected cells, that are able to efficiently convert extracellular ATP in anti-inflammatory molecule adenosine (Fig 4), were able to up-regulate both the *Tnfaip3* and *Nemo* anti-apoptotic genes as a consequence of the constitutive expression and activity of hENTPD1 and hE5NT genes. On the contrary, in presence of TNF-α alone, control cells are able to up-regulate the *Tnfaip3* gene that is not protective enough because the TNF-α exposure is not transient. Furthermore, the control cells survival is further compromised when ATP is added to the TNF-α treatment because control cells did not constitutively express any protective gene. In summary, pCX-TRI-2A-transfected cells up-regulated key regulators of TNF-α signalling pathway, namely *Nemo* and *Tnfaip3*, which induced the Nf-kB-mediated pro-survival effects in pCX-TRI-2A-transfected cells exposed to TNF-α injury.

In conclusion, this study demonstrated, for the first time, the anti-inflammatory and anti-apoptotic effects of a combination of three human genes simultaneously expressed in murine cells via an F2A system-based multicistronic approach. The protective effects against TNF-α-induced cytotoxicity and cell death, mediated by HO-1, ENTPD1 and E5NT genes were better observed in cells expressing the combination of genes as compared to cells expressing each single gene and the effect was further improved by administrating enzymatic substrates of the human genes to the cells. Moreover, a gene expression analyses suggested that the expression of the three genes has a role in modulating key downstream regulators of TNF-α signalling pathway, as *Nemo* and *Tnfaip3*, that promoted pro-survival phenotype in TNF-α injured cells. Given these anti-inflammatory and anti-apoptotic effects, we suggest to use pCX-TRI-2A in order to produce multi-gene transgenic pigs, whose organs and cells could be tested in pre-clinical xenotransplantation models. Future works will be aimed to investigate the ENTPD1-mediated protection against the coagulation and thrombotic disorders in xenotransplantation as well as the immunosuppressive effects derived by the ENTPD1/E5NT-mediated production of adenosine.

## Supporting Information

S1 Fig PCR analysis on genomic DNA. PCR were performed on 75ng of genomic DNA extracted from WT and pCX-TRI-2A-transfected cells. Two primer pairs were used: 5’HO1 fw (CTGGAGGAGGAGATTGAGCG) / 2A rev (CGCCAACTTGAGAAGGTCAAAA) pair that covers the region from 5’ of hHO1 CDS to the first 2A sequence; intern E5NT fw (TGTTGGTGATGAAGTTGTGG) / 2A rev (CGCCAACTTGAGAAGGTCAAAA) pair that covers the region from hE5NT CDS to the second 2A sequence. Results show the presence of amplicons with expected size, respectively 753bp for hHO-1 and 1297bp for hCD73. As positive control 75ng of gDNA from WT cells mixed with 10^2^ copies of pCX-TRI-2A plasmid were used.
(TIF)

S2 Fig PCR analysis on RNA. End-point PCR were performed on 25ng of cDNA retrotranscribed (+) from total RNA extracted from WT and pCX-TRI-2A-transfected cells. Two primer pairs were used: 5’HO1 fw (CTGGAGGAGGAGATTGAGCG) / 2A rev (CGCCAACTTGAGAAGGTCAAAA) pair that covers the region from 5’ of hHO1 CDS to the first 2A sequence; intern E5NT fw (TGTTGGTGATGAAGTTGTGG) / 2A rev (CGCCAACTTGAGAAGGTCAAAA) pair that covers the region from hE5NT CDS to the second 2A sequence. Results show the presence of amplicons with expected size, respectively 753bp for hHO-1 and 1297bp for hCD73. 10^3^ copies of plasmids diluted into 25ng of WT cDNA were amplified as positive controls of PCR reaction. Phosphoglycerate kinase (PGK) housekeeping end-point PCR were performed using PGK1-HK-fw (GTATCCCTATGCCTGACAAGT) / PGK1-HK-rev (TTCCCTTCTTCCTCCACAT) primers pair, on 25ng of cDNA from WT and TG cells. Expected size band, 187bp, is visible in RT+ of each type of cells.
(TIF)

S3 Fig Single-gene transfected cells expression analysis. Appropriate single gene-vectors have been produced as control of transfection as well as to investigate the contribution of each gene in the downregulation of the inflammatory response. pCX-E5NT and pCX-hENTPD1 transfected cells were sorted and analyzed for hE5NT and hENTPD1 expression respectively. pCX-HO1 transfected cells were sorted and analyzed on the basis of EGFP expression. After sorting each population count at least 98% of cells expressing the exogenous protein. WT and mock-transfected cells showed no expression of any of the three human proteins.
(TIF)

S4 Fig Propidium Iodide incorporation assay. 1×106 cells were seeded in 10 ml culture petri and treated with medium containing TNF-α (50 ng/ml) alone or with TNF-α (50 ng/ml), hemin (20 μM) and ATP (200 μM) for 24, 48 and 72 hours. Untreated cells were also cultured as a control of basal level of cell death. Cell death was detected, at each time point, using propidium iodide (PI, Sigma Aldrich) influx evaluation. At the end of treatment, the cells were harvested by centrifugation and suspended in PBS. Subsequently, the cells were incubated with 2 μg/mL of propidium iodide (PI) in the dark for 15 min at room temperature immediately before cytometric evaluation on FACSARIA flow cytometer (Becton Dickinson, San Jose, CA). PI incorporation was detected by red fluorescence on a log scale and cell death percentages were calculated on PI+cells combined with the scatter (FSC) by subtracting the % of untreated cells at each condition. Data were collected (at least 50,000 events) and analyzed using DIVA software (Becton Dickinson) and FlowJo software.
(TIF)

S5 Fig RT^2^ Profiler PCR array of TNF-α signaling genes in control (Ctrl) cells. The 3D Profile showed the fold difference in expression of each gene between control cells treated with TNF-α 50 ng/ml in combination with hemin 20 μM and ATP 200 μM (test sample) at 16h and untreated cells (UT, control sample). Columns pointing up (with z-axis values > 1) indicate an up-regulation of gene expression, while columns pointing down (with z-axis values < 1) indicate a down-regulation of gene expression in the test sample relative to the control sample.
(TIF)

S6 Fig RT^2^ Profiler PCR array of TNF-α signaling genes in pCX-TRI-2A-transfected cells. The 3D Profile showed the fold difference in expression of each gene between pCX-TRI-2A-transfected cells treated with TNF-α 50 ng/ml in combination with hemin 20 μM and ATP 200 μM (test sample) at 16h and untreated cells (UT, control sample). Columns pointing up (with z-axis values > 1) indicate an up-regulation of gene expression, while columns pointing down (with z-axis values < 1) indicate a down-regulation of gene expression in the test sample relative to the control sample.
(TIF)

S1 Table Oligonucleotides used for real time PCR experiments. The primers name and sequences are reported. The melting temperature (Tm) is indicated in Celsius grade. Primers for *Ikbkg* genes were designed by using Primer3 software (Untergasser A, *et al*. Primer3Plus, an enhanced web interface to Primer3. *Nucl*. *Acids Res*. 2007 35: W71-4). Primer sequences for *Gapdh* gene were recovered from PrimerBank repository (Spandidos A, *et* al. PrimerBank: a resource of human and mouse PCR primer pairs for gene expression detection and quantification. *Nucl*. *Acids Res*. 2010 38: D792-9).
(DOCX)

S2 Table TNF-α pathway-related genes differently modulated between pCX-TRI-2A-transfected cells and Ctrl cells. Genes resulted differently modulated between pCX-TRI-2A-transfected cells and Ctrl cells are listed in table. The genes name and a brief description of the related proteins function are reported. Values are expressed as fold difference in gene expression by comparing each cell lines treated with TNF-α 50 + hemin 20 μM + ATP 200 μM to the respective untreated cells.
(DOCX)

## Abbreviations

F2A: Foot and Mouth Disease Virus 2A sequence
hHO-1: human heme oxygenase 1
hE5NT: human ecto-5’-nucleotidase
hENTPD1: human ecto-nucleoside triphosphate diphosphohydrolases
NF-kB: nuclear factor kB
TNF-α: tumor necrosis factor-alpha
TNFR1: TNF receptor 1
TNFR2: TNF receptor 2

## References

[1] Kriz A, Schmid K, Baumgartner N, Ziegler U, Berger I, Ballmer-Hofer K, et al A plasmid-based multigene expression system for mammalian cells. Nat Comms. 2010;1: 120 10.1038/ncomms1120 21081918

[2] Luke GA, Ryan MD. The protein coexpression problem in biotechnology and biomedicine: virus 2A and 2A-like sequences provide a solution. Future Virology. 2013;8: 983–996. 10.2217/fvl.13.82

[3] Chan HY, V S, Xing X, Kraus P, Yap SP, Ng P, et al Comparison of IRES and F2A-based locus-specific multicistronic expression in stable mouse lines. PLoS ONE. Public Library of Science; 2011;6: e28885 10.1371/journal.pone.0028885 22216134 PMC3244433

[4] de Felipe P, Luke GA, Hughes LE, Gani D, Halpin C, Ryan MD. E unum pluribus: multiple proteins from a self-processing polyprotein. Trends Biotechnol. 2006;24: 68–75. 10.1016/j.tibtech.2005.12.006 16380176

[5] Yang S, Cohen CJ, Peng PD, Zhao Y, Cassard L, Yu Z, et al Development of optimal bicistronic lentiviral vectors facilitates high-level TCR gene expression and robust tumor cell recognition. Gene Ther. 2008;15: 1411–1423. 10.1038/gt.2008.90 18496571 PMC2684456

[6] Fang J, Qian J-J, Yi S, Harding TC, Tu GH, VanRoey M, et al Stable antibody expression at therapeutic levels using the 2A peptide. Nat Biotechnol. 2005;23: 584–590. 10.1038/nbt1087 15834403

[7] Griesemer A, Yamada K, Sykes M. Xenotransplantation: immunological hurdles and progress toward tolerance. Immunol Rev. 2014;258: 241–258. 10.1111/imr.12152 24517437 PMC4023346

[8] Gock H, Nottle M, Lew AM, D'Apice AJF, Cowan P. Genetic modification of pigs for solid organ xenotransplantation. Transplant Rev (Orlando). 2011;25: 9–20. 10.1016/j.trre.2010.10.001 21126659

[9] Robson SC, Schulte am Esch J, Bach FH. Factors in xenograft rejection. Ann N Y Acad Sci. 1999;875: 261–276. 10415573 10.1111/j.1749-6632.1999.tb08509.x

[10] Mori DN, Kreisel D, Fullerton JN, Gilroy DW, Goldstein DR. Inflammatory triggers of acute rejection of organ allografts. Immunol Rev. 2014;258: 132–144. 10.1111/imr.12146 24517430 PMC3939032

[11] Charniot J-C, Bonnefont-Rousselot D, Albertini J-P, Zerhouni K, Dever S, Richard I, et al Oxidative stress implication in a new ex-vivo cardiac concordant xenotransplantation model. Free Radic Res. 2007;41: 911–918. 10.1080/10715760701429775 17654048

[12] Ngo BT-T, Beiras-Fernandez A, Hammer C, Thein E. Hyperacute rejection in the xenogenic transplanted rat liver is triggered by the complement system only in the presence of leukocytes and free radical species. Xenotransplantation. 2013;20: 177–187. 10.1111/xen.12035 23656281

[13] Gozzelino R, Jeney V, Soares MP. Mechanisms of cell protection by heme oxygenase-1. Annu Rev Pharmacol Toxicol. 2010;50: 323–354. 10.1146/annurev.pharmtox.010909.105600 20055707

[14] Öllinger R, Pratschke J. Role of heme oxygenase-1 in transplantation. Transpl Int. Blackwell Publishing Ltd; 2010;23: 1071–1081. 10.1111/j.1432-2277.2010.01158.x 20819190

[15] Wegiel B, Nemeth Z, Correa-Costa M, Bulmer AC, Otterbein LE. Heme Oxygenase-1: A Metabolic Nike. Antioxidants & Redox Signaling. 2014;: 140227074814002. 10.1089/ars.2013.5667 24180257 PMC3961788

[16] Lavitrano M, Smolenski RT, Musumeci A, Maccherini M, Slominska EM, Di Florio E, et al Carbon monoxide improves cardiac energetics and safeguards the heart during reperfusion after cardiopulmonary bypass in pigs. FASEB J. 2004;18: 1093–1095. 10.1096/fj.03-0996fje 15132974

[17] Smolenski RT, Khalpey Z, Osborne FN, Yuen A, Slominska EM, Lipiński M, et al Species differences of endothelial extracellular nucleotide metabolism and its implications for xenotransplantation. Pharmacol Rep. 2006;58 Suppl: 118–125. 17332681

[18] Parmely MJ, Zhou WW, Edwards CK, Borcherding DR, Silverstein R, Morrison DC. Adenosine and a related carbocyclic nucleoside analogue selectively inhibit tumor necrosis factor-alpha production and protect mice against endotoxin challenge. J Immunol. 1993;151: 389–396. 8326132

[19] Lappin D, Whaley K. Adenosine A2 receptors on human monocytes modulate C2 production. Clin Exp Immunol. 1984;57: 454–460. 6088136 PMC1536127

[20] Cronstein BN, Kramer SB, Weissmann G, Hirschhorn R. Adenosine: a physiological modulator of superoxide anion generation by human neutrophils. J Exp Med. 1983;158: 1160–1177. 6311934 10.1084/jem.158.4.1160 PMC2187367

[21] Antonioli L, Pacher P, Vizi ES, Haskó G. CD39 and CD73 in immunity and inflammation. Trends in molecular medicine. 2013;19: 355–367. 10.1016/j.molmed.2013.03.005 23601906 PMC3674206

[22] Koszalka P, Ozüyaman B, Huo Y, Zernecke A, Flögel U, Braun N, et al Targeted disruption of cd73/ecto-5'-nucleotidase alters thromboregulation and augments vascular inflammatory response. Circ Res. 2004;95: 814–821. 10.1161/01.RES.0000144796.82787.6f 15358667

[23] Enjyoji K, Sévigny J, Lin Y, Frenette PS, Christie PD, Esch JS, et al Targeted disruption of cd39/ATP diphosphohydrolase results in disordered hemostasis and thromboregulation. Nat Med. 1999;5: 1010–1017. 10.1038/12447 10470077

[24] Osborne FN, Kalsi KK, Lawson C, Lavitrano M, Yacoub MH, Rose ML, et al Expression of human ecto-5'-nucleotidase in pig endothelium increases adenosine production and protects from NK cell-mediated lysis. Am J Transplant. 2005;5: 1248–1255. 10.1111/j.1600-6143.2005.00868.x 15888028

[25] Cai M, Huttinger ZM, He H, Zhang W, Li F, Goodman LA, et al Transgenic over expression of ectonucleotide triphosphate diphosphohydrolase-1 protects against murine myocardial ischemic injury. J Mol Cell Cardiol. 2011;51: 927–935. 10.1016/j.yjmcc.2011.09.003 21939667 PMC3245748

[26] Zelová H, Hošek J. TNF-α signalling and inflammation: interactions between old acquaintances. Inflamm Res. SP Birkhäuser Verlag Basel; 2013;62: 641–651. 10.1007/s00011-013-0633-0 23685857

[27] Shuh M, Bohorquez H, Loss GE, Cohen AJ. Tumor Necrosis Factor-α: Life and Death of Hepatocytes During Liver Ischemia/Reperfusion Injury. Ochsner J. 2013;13: 119–130. 23531747 PMC3603175

[28] Ahn J, Kim J. Mechanisms and consequences of inflammatory signaling in the myocardium. Curr Hypertens Rep. Current Science Inc; 2012;14: 510–516. 10.1007/s11906-012-0309-0 22986910

[29] Cooper DKC, Ekser B, Burlak C, Ezzelarab M, Hara H, Paris L, et al Clinical lung xenotransplantation—what donor genetic modifications may be necessary? Xenotransplantation. 2012;19: 144–158. 10.1111/j.1399-3089.2012.00708.x 22702466 PMC3775598

[30] Speeckaert MM, Speeckaert R, Laute M, Vanholder R, Delanghe JR. Tumor necrosis factor receptors: biology and therapeutic potential in kidney diseases. Am J Nephrol. 2012;36: 261–270. 10.1159/000342333 22965073

[31] Ryan MD, Drew J. Foot-and-mouth disease virus 2A oligopeptide mediated cleavage of an artificial polyprotein. EMBO J. 1994;13: 928–933. 8112307 10.1002/j.1460-2075.1994.tb06337.x PMC394894

[32] De Giorgi M, Cinti A, Pelikant-Malecka I, Chisci E, Lavitrano M, Giovannoni R, et al Co-expression of functional human Heme Oxygenase 1, Ecto-5′-Nucleotidase and ecto-nucleoside triphosphate diphosphohydrolase-1 by “self-cleaving” 2A peptide system. Plasmid. 2015;79: 22–29. 10.1016/j.plasmid.2015.03.004 25779031

[33] Okabe M, Ikawa M, Kominami K, Nakanishi T, Nishimune Y. “Green mice” as a source of ubiquitous green cells. FEBS Letters. 1997;407: 313–319. 10.1016/S0014-5793(97)00313-X 9175875

[34] Schneider CA, Rasband WS, Eliceiri KW. NIH Image to ImageJ: 25 years of image analysis. Nat Methods. 2012;9: 671–675. 10.1038/nmeth.2089 22930834 PMC5554542

[35] Klemz R, Mashreghi M-F, Spies C, Volk H-D, Kotsch K. Free Radical Biology & Medicine. Free Radic Biol Med. Elsevier Inc; 2009;46: 305–311. 10.1016/j.freeradbiomed.2008.10.044 19038332

[36] De Giorgi M, Pelikant-Malecka I, Sielicka A, Slominska EM, Giovannoni R, Cinti A, et al Functional Analysis of Expression of Human Ecto-Nucleoside Triphosphate Diphosphohydrolase-1 and/or Ecto-5'-Nucleotidase in Pig Endothelial Cells. Nucleosides Nucleotides Nucleic Acids. 2014;33: 313–318. 10.1080/15257770.2014.896466 24940685

[37] Smolenski RT, Lachno DR, Ledingham SJ, Yacoub MH. Determination of sixteen nucleotides, nucleosides and bases using high-performance liquid chromatography and its application to the study of purine metabolism in hearts for transplantation. J Chromatogr. 1990;527: 414–420. 2387888 10.1016/s0378-4347(00)82125-8

[38] Rothwell DG, Crossley R, Bridgeman JS, Sheard V, Zhang Y, Sharp TV, et al Functional expression of secreted proteins from a bicistronic retroviral cassette based on foot-and-mouth disease virus 2A can be position dependent. Hum Gene Ther. 2010;21: 1631–1637. 10.1089/hum.2009.197 20528679

[39] Cabal-Hierro L, Lazo PS. Signal transduction by tumor necrosis factor receptors. Cell Signal. 2012;24: 1297–1305. 10.1016/j.cellsig.2012.02.006 22374304

[40] Micheau O, Tschopp J. Induction of TNF receptor I-mediated apoptosis via two sequential signaling complexes. Cell. 2003;114: 181–190. 12887920 10.1016/s0092-8674(03)00521-x

[41] Kim S-J, Eum H-A, Billiar TR, Lee S-M. Role of heme oxygenase 1 in TNF/TNF receptor-mediated apoptosis after hepatic ischemia/reperfusion in rats. Shock. 2013;39: 380–388. 10.1097/SHK.0b013e31828aab7f 23423194

[42] Crikis S, Lu B, Murray-Segal LM, Selan C, Robson SC, D'Apice AJF, et al Transgenic overexpression of CD39 protects against renal ischemia-reperfusion and transplant vascular injury. Am J Transplant. Blackwell Publishing Inc; 2010;10: 2586–2595. 10.1111/j.1600-6143.2010.03257.x 20840479 PMC5472986

[43] Pujari R, Hunte R, Khan WN, Shembade N. A20-mediated negative regulation of canonical NF-κB signaling pathway. Immunol Res. Springer US; 2013;57: 166–171. 10.1007/s12026-013-8463-2 24242761

[44] Opipari AW, Hu HM, Yabkowitz R, Dixit VM. The A20 zinc finger protein protects cells from tumor necrosis factor cytotoxicity. J Biol Chem. 1992;267: 12424–12427. 1618749

[45] Fei D, Meng X, Zhao M, Kang K, Tan G, Pan S, et al Enhanced induction of heme oxygenase-1 suppresses thrombus formation and affects the protein C system in sepsis. Transl Res. 2012;159: 99–109. 10.1016/j.trsl.2011.10.009 22243794

[46] Paine A, Eiz-Vesper B, Blasczyk R, Immenschuh S. Signaling to heme oxygenase-1 and its anti-inflammatory therapeutic potential. Biochem Pharmacol. 2010;80: 1895–1903. 10.1016/j.bcp.2010.07.014 20643109

[47] Wheeler DG, Joseph ME, Mahamud SD, Aurand WL, Mohler PJ, Pompili VJ, et al Transgenic swine: Expression of human CD39 protects against myocardial injury. J Mol Cell Cardiol. 2012;52: 958–961. 10.1016/j.yjmcc.2012.01.002 22269791 PMC3327755

[48] Grünewald JK, Ridley AJ. CD73 represses pro-inflammatory responses in human endothelial cells. J Inflamm (Lond). BioMed Central Ltd; 2010;7: 10 10.1186/1476-9255-7-10 20181103 PMC2833156

[49] Bach FH, Ferran C, Hechenleitner P, Mark W, Koyamada N, Miyatake T, et al Accommodation of vascularized xenografts: Expression of ''protective genes“” by donor endothelial cells in a host Th2 cytokine environment. Nat Med. 1997;3: 196–204. 9018239 10.1038/nm0297-196

[50] Soares MP, Lin Y, Anrather J, Csizmadia E, Takigami K, Sato K, et al Expression of heme oxygenase-1 can determine cardiac xenograft survival. Nat Med. 1998;4: 1073–1077. 10.1038/2063 9734404

[51] Tabata T, de Perrot M, Keshavjee S, Liu M, Downey GP, Waddell TK. Accommodation after lung xenografting from hamster to rat. Transplantation. 2003;75: 607–612. 10.1097/01.TP.0000053353.03389.1C 12640297

[52] Dwyer KM, Robson SC, Nandurkar HH, Campbell DJ, Gock H, Murray-Segal LJ, et al Thromboregulatory manifestations in human CD39 transgenic mice and the implications for thrombotic disease and transplantation. J Clin Invest. 2004;113: 1440–1446. 10.1172/JCI19560 15146241 PMC406523

[53] Dwyer KM, Mysore TB, Crikis S, Robson SC, Nandurkar H, Cowan PJ, et al The transgenic expression of human CD39 on murine islets inhibits clotting of human blood. Transplantation. 2006;82: 428–432. 10.1097/01.tp.0000229023.38873.c0 16906044

[54] Iwase H, Ezzelarab M, Ekser B, Cooper DKC. The role of platelets in coagulation dysfunction in xenotransplantation, and therapeutic options. Xenotransplantation. 2014;21: 201–220. 10.1111/xen.12085 24571124

[55] Fisicaro N, Londrigan SL, Brady JL, Salvaris E, Nottle MB, O’Connell PJ, et al Versatile co-expression of graft-protective proteins using 2A-linked cassettes. Xenotransplantation. Blackwell Publishing Ltd; 2011;18: 121–130. 10.1111/j.1399-3089.2011.00631.x 21496119 PMC3785253

[56] Yin W, Xiang P, Li Q. Investigations of the effect of DNA size in transient transfection assay using dual luciferase system. Analytical Biochemistry. 2005;346: 289–294. 10.1016/j.ab.2005.08.029 16213455

[57] Klemz R, Mashreghi M-F, Spies C, Volk H-D, Kotsch K. Amplifying the fluorescence of bilirubin enables the real-time detection of heme oxygenase activity. Free Radic Biol Med. 2009;46: 305–311. 10.1016/j.freeradbiomed.2008.10.044 19038332

[58] Bradley JR. TNF-mediated inflammatory disease. J Pathol. John Wiley & Sons, Ltd; 2008;214: 149–160. 10.1002/path.2287 18161752

[59] Rath PC, Aggarwal BB. TNF-induced signaling in apoptosis. J Clin Immunol. 1999;19: 350–364. 10634209 10.1023/a:1020546615229

[60] Brouard S, Otterbein LE, Anrather J, Tobiasch E, Bach FH, Choi AM, et al Carbon monoxide generated by heme oxygenase 1 suppresses endothelial cell apoptosis. J Exp Med. 2000;192: 1015–1026. 11015442 10.1084/jem.192.7.1015 PMC2193315

[61] Akamatsu Y, Haga M, Tyagi S, Yamashita K, Graca-Souza AV, Ollinger R, et al Heme oxygenase-1-derived carbon monoxide protects hearts from transplant-associated ischemia reperfusion injury. FASEB J. Federation of American Societies for Experimental Biology; 2004;18: 771–. 10.1096/fj.03-0921fje 14977880

[62] Sato K, Balla J, Otterbein L, Smith RN, Brouard S, Lin Y, et al Carbon monoxide generated by heme oxygenase-1 suppresses the rejection of mouse-to-rat cardiac transplants. J Immunol. American Association of Immunologists; 2001;166: 4185–4194. 10.4049/jimmunol.166.6.4185 11238670

[63] Fondevila C, Shen X-D, Tsuchiyashi S, Yamashita K, Csizmadia E, Lassman C, et al Biliverdin therapy protects rat livers from ischemia and reperfusion injury. Hepatology. 2004;40: 1333–1341. 10.1002/hep.20480 15565657

[64] Wang HJ, Lee SS, Dell'Agnello C, Tchipashvili V, D'Avilla J, Czismadia E, et al Bilirubin can induce tolerance to islet allografts. Endocrinology. 2006;147: 762–768. 10.1210/en.2005-0632 16254033

[65] Oellinger R, Wang H, Yamashita K, Wegiel B, Thomas M, Margreiter R, et al Therapeutic applications of bilirubin and biliverdin in transplantation. Antioxidants & Redox Signaling. 2007;9: 2175–2185. 10.1089/ars.2007.1807 17919067

[66] Fang J, Qin H, Seki T, Nakamura H, Tsukigawa K, Shin T, et al Therapeutic potential of pegylated hemin for reactive oxygen species-related diseases via induction of heme oxygenase-1: results from a rat hepatic ischemia/reperfusion injury model. Journal of Pharmacology and Experimental Therapeutics. American Society for Pharmacology and Experimental Therapeutics; 2011;339: 779–789. 10.1124/jpet.111.185348 21890508

[67] Zhen-Wei X, Jian-Le S, Qi Q, Wen-Wei Z, Xue-Hong Z, Zi-Li Z. Heme oxygenase-1 improves the survival of discordant cardiac xenograft through its anti-inflammatory and anti-apoptotic effects. Pediatr Transplant. Blackwell Publishing Ltd; 2007;11: 850–859. 10.1111/j.1399-3046.2007.00701.x 17976119

[68] Delikouras A, Fairbanks LD, Simmonds AH, Lechler RI, Dorling A. Endothelial cell cytoprotection induced in vitro by allo- or xenoreactive antibodies is mediated by signaling through adenosine A2 receptors. Eur J Immunol. 2003;33: 3127–3135. 10.1002/eji.200323566 14579281

[69] Eltzschig HK, Bonney SK, Eckle T. Attenuating myocardial ischemia by targeting A2B adenosine receptors. Trends in molecular medicine. 2013;19: 345–354. 10.1016/j.molmed.2013.02.005 23540714 PMC3674126

[70] Li J, Yin Q, Wu H. Structural basis of signal transduction in the TNF receptor superfamily. Adv Immunol. 2013;119: 135–153. 10.1016/B978-0-12-407707-2.00005-9 23886067 PMC3781945

[71] Israël A. The IKK complex, a central regulator of NF-kappaB activation. Cold Spring Harb Perspect Biol. 2010;2: a000158 10.1101/cshperspect.a000158 20300203 PMC2829958

