# Supplementary figures and images for: Simultaneous Overexpression of Functional Human HO-1, E5NT and ENTPD1 Protects Murine Fibroblasts against TNF-α-Induced Injury In Vitro
Source: PLoS One. 2015 Oct 29;10(10):e0141933. doi: 10.1371/journal.pone.0141933 (PMC4626094; doi:10.1371/journal.pone.0141933)

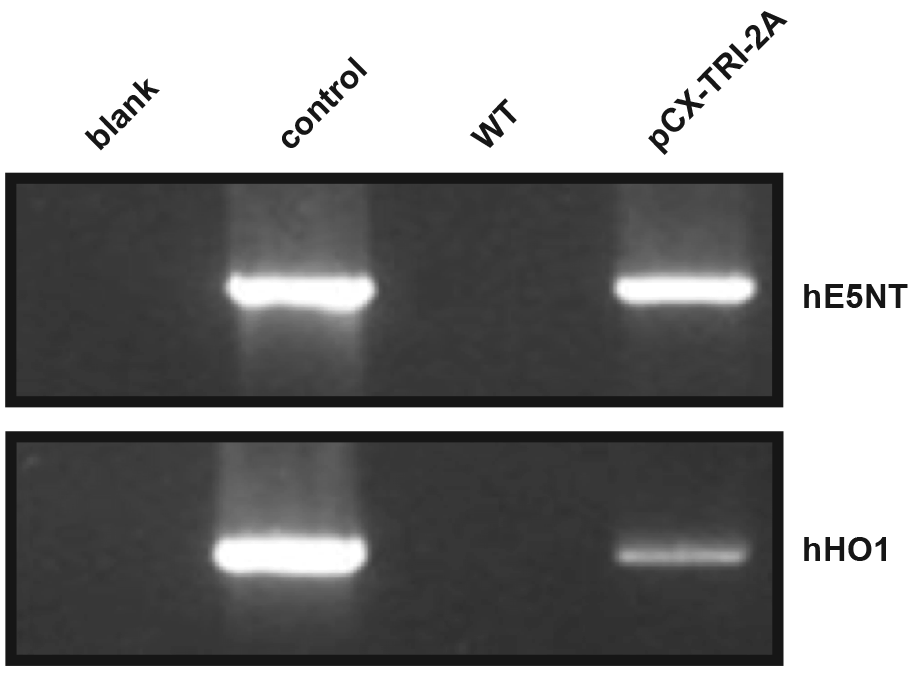

Supplement: S1 Fig — PCR were performed on 75ng of genomic DNA extracted from WT and pCX-TRI-2A-transfected cells. Two primer pairs were used: 5’HO1 fw (CTGGAGGAGGAGATTGAGCG) / 2A rev (CGCCAACTTGAGAAGGTCAAAA) pair that covers the region from 5’ of hHO1 CDS to the first 2A sequence; intern E5NT fw (TGTTGGTGATGAAGTTGTGG) / 2A rev (CGCCAACTTGAGAAGGTCAAAA) pair that covers the region from hE5NT CDS to the second 2A sequence. Results show the presence of amplicons with expected size, respectively 753bp for hHO-1 and 1297bp for hCD73. As positive control 75ng of gDNA from WT cells mixed with 102 copies of pCX-TRI-2A plasmid were used. (TIF) [file pone.0141933.s001.tif]

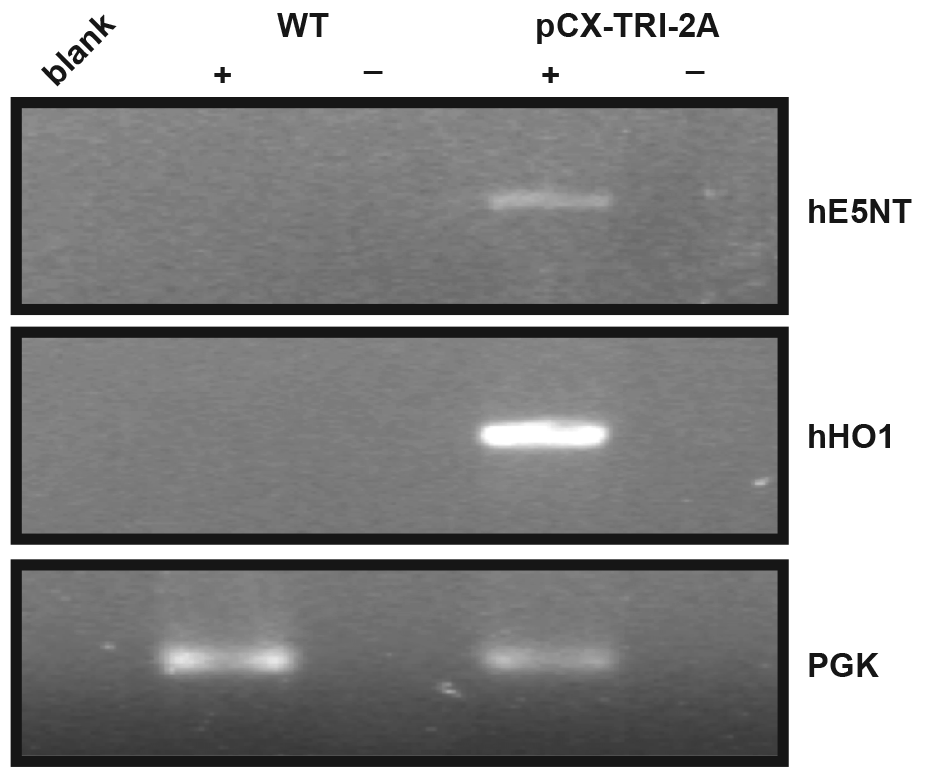

Supplement: S2 Fig — End-point PCR were performed on 25ng of cDNA retrotranscribed (+) from total RNA extracted from WT and pCX-TRI-2A-transfected cells. Two primer pairs were used: 5’HO1 fw (CTGGAGGAGGAGATTGAGCG) / 2A rev (CGCCAACTTGAGAAGGTCAAAA) pair that covers the region from 5’ of hHO1 CDS to the first 2A sequence; intern E5NT fw (TGTTGGTGATGAAGTTGTGG) / 2A rev (CGCCAACTTGAGAAGGTCAAAA) pair that covers the region from hE5NT CDS to the second 2A sequence. Results show the presence of amplicons with expected size, respectively 753bp for hHO-1 and 1297bp for hCD73. 103 copies of plasmids diluted into 25ng of WT cDNA were amplified as positive controls of PCR reaction. Phosphoglycerate kinase (PGK) housekeeping end-point PCR were performed using PGK1-HK-fw (GTATCCCTATGCCTGACAAGT) / PGK1-HK-rev (TTCCCTTCTTCCTCCACAT) primers pair, on 25ng of cDNA from WT and TG cells. Expected size band, 187bp, is visible in RT+ of each type of cells. (TIF) [file pone.0141933.s002.tif]

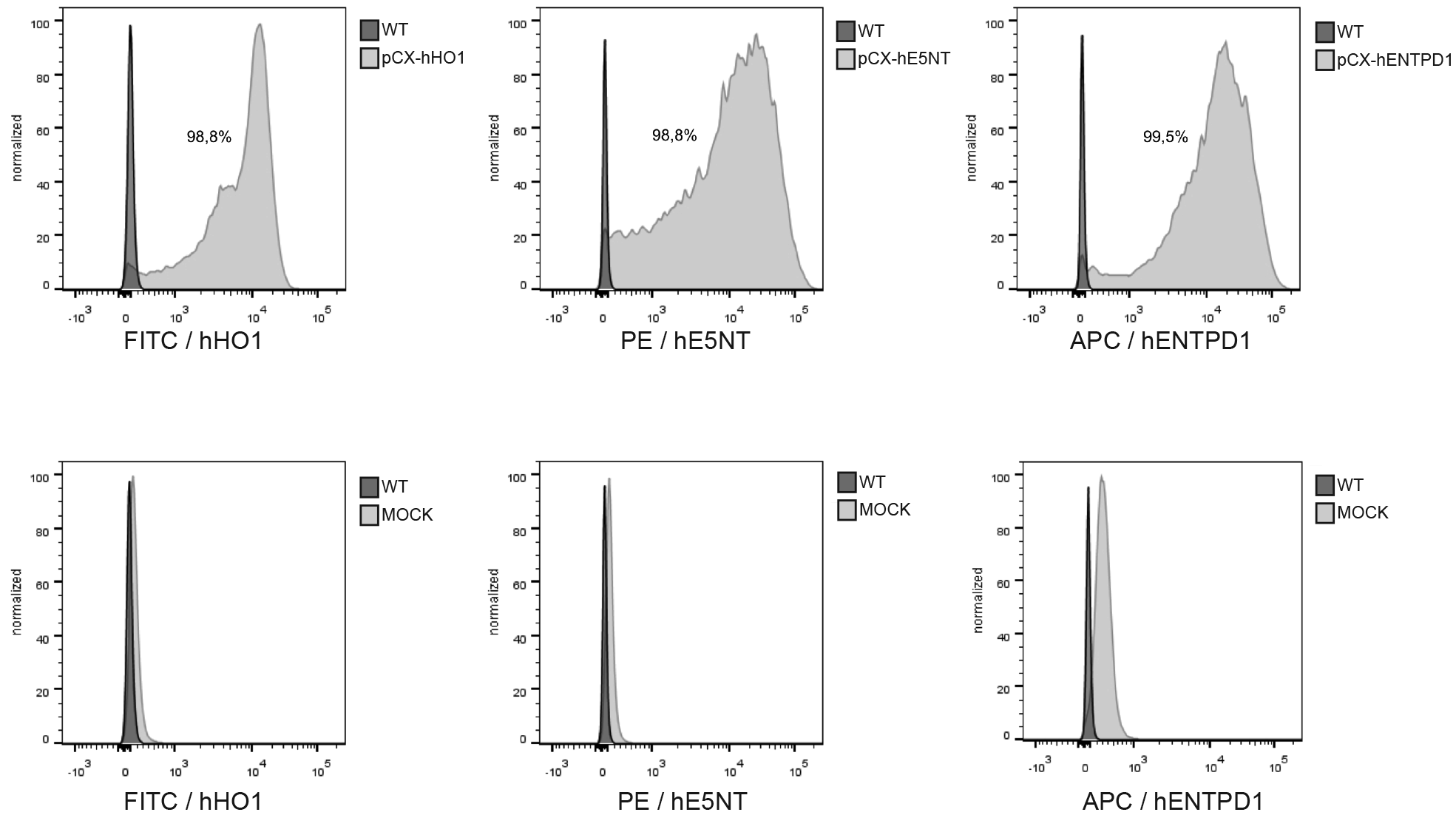

Supplement: S3 Fig — Appropriate single gene-vectors have been produced as control of transfection as well as to investigate the contribution of each gene in the downregulation of the inflammatory response. pCX-E5NT and pCX-hENTPD1 transfected cells were sorted and analyzed for hE5NT and hENTPD1 expression respectively. pCX-HO1 transfected cells were sorted and analyzed on the basis of EGFP expression. After sorting each population count at least 98% of cells expressing the exogenous protein. WT and mock-transfected cells showed no expression of any of the three human proteins. (TIF) [file pone.0141933.s003.tif]

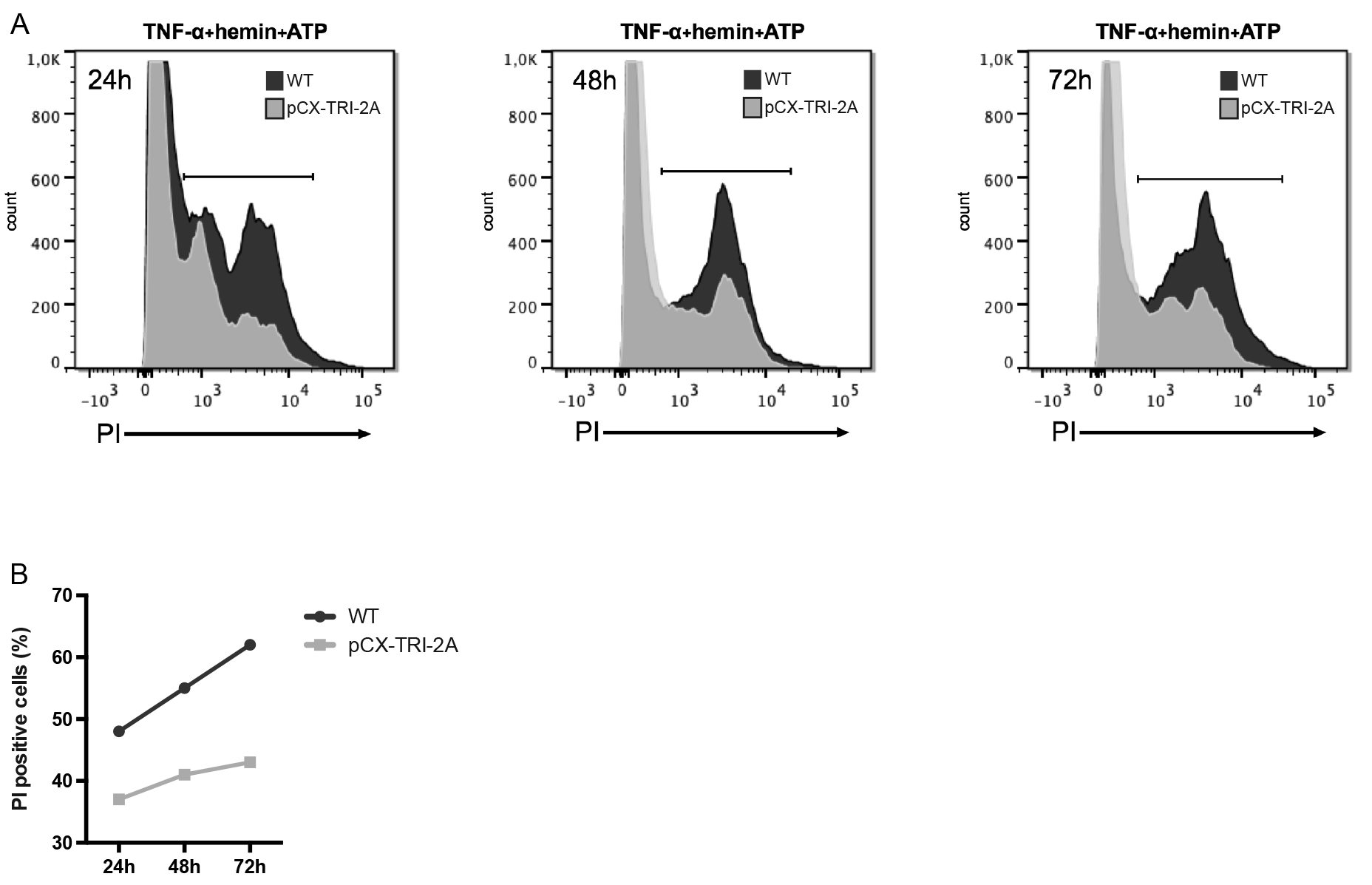

Supplement: S4 Fig — 1×10^6^ cells were seeded in 10 ml culture petri and treated with medium containing TNF-α (50 ng/ml) alone or with TNF-α (50 ng/ml), hemin (20 μM) and ATP (200 μM) for 24, 48 and 72 hours. Untreated cells were also cultured as a control of basal level of cell death. Cell death was detected, at each time point, using propidium iodide (PI, Sigma Aldrich) influx evaluation. At the end of treatment, the cells were harvested by centrifugation and suspended in PBS. Subsequently, the cells were incubated with 2 μg/mL of propidium iodide (PI) in the dark for 15 min at room temperature immediately before cytometric evaluation on FACSARIA flow cytometer (Becton Dickinson, San Jose, CA). PI incorporation was detected by red fluorescence on a log scale and cell death percentages were calculated on PI+cells combined with the scatter (FSC) by subtracting the % of untreated cells at each condition. Data were collected (at least 50,000 events) and analyzed using DIVA software (Becton Dickinson) and FlowJo software. (TIF) [file pone.0141933.s004.tif]

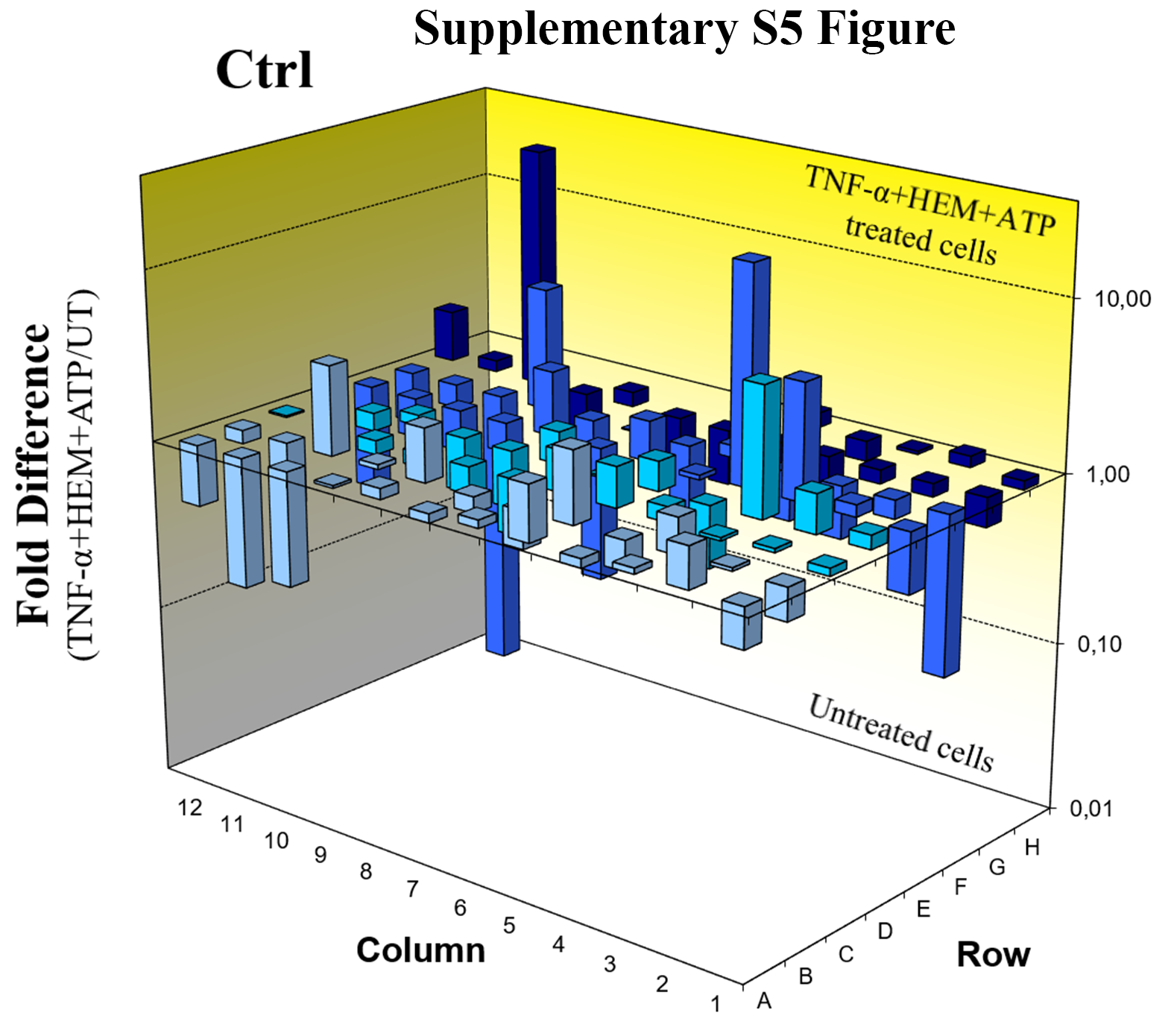

Supplement: S5 Fig — The 3D Profile showed the fold difference in expression of each gene between control cells treated with TNF-α 50 ng/ml in combination with hemin 20 μM and ATP 200 μM (test sample) at 16h and untreated cells (UT, control sample). Columns pointing up (with z-axis values > 1) indicate an up-regulation of gene expression, while columns pointing down (with z-axis values < 1) indicate a down-regulation of gene expression in the test sample relative to the control sample. (TIF) [file pone.0141933.s005.tif]

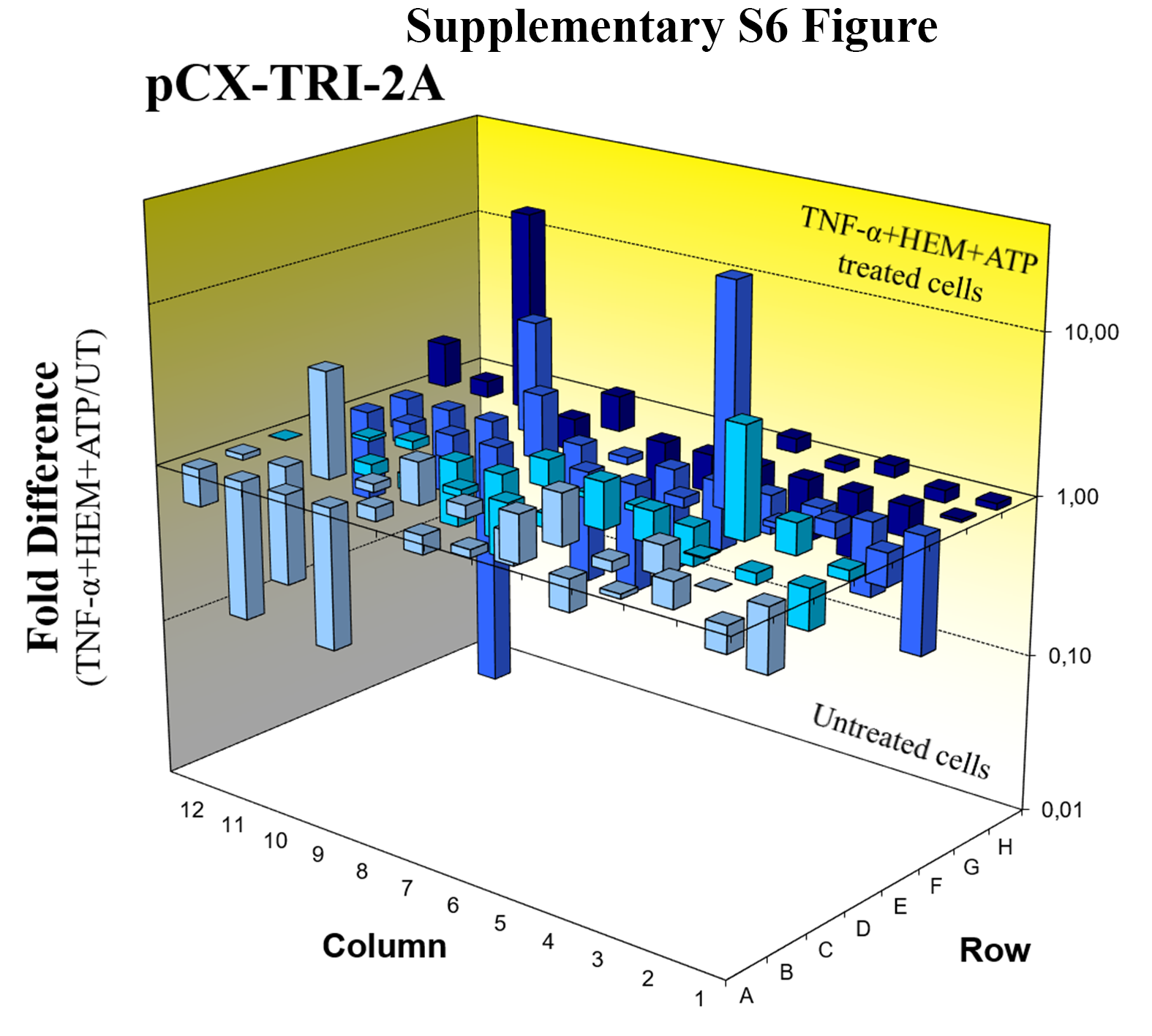

Supplement: S6 Fig — The 3D Profile showed the fold difference in expression of each gene between pCX-TRI-2A-transfected cells treated with TNF-α 50 ng/ml in combination with hemin 20 μM and ATP 200 μM (test sample) at 16h and untreated cells (UT, control sample). Columns pointing up (with z-axis values > 1) indicate an up-regulation of gene expression, while columns pointing down (with z-axis values < 1) indicate a down-regulation of gene expression in the test sample relative to the control sample. (TIF) [file pone.0141933.s006.tif]
