# Supplementary material for: Simultaneous Overexpression of Functional Human HO-1, E5NT and ENTPD1 Protects Murine Fibroblasts against TNF-α-Induced Injury In Vitro
Source: PLoS One. 2015 Oct 29;10(10):e0141933. doi: 10.1371/journal.pone.0141933 (PMC4626094; doi:10.1371/journal.pone.0141933)
Supplement: S1 Table — The primers name and sequences are reported. The melting temperature (Tm) is indicated in Celsius grade. Primers for Ikbkg genes were designed by using Primer3 software (Untergasser A, et al. Primer3Plus, an enhanced web interface to Primer3. Nucl. Acids Res. 2007 35: W71-4). Primer sequences for Gapdh gene were recovered from PrimerBank repository (Spandidos A, et al. PrimerBank: a resource of human and mouse PCR primer pairs for gene expression detection and quantification. Nucl. Acids Res. 2010 38: D792-9). (DOCX) [file pone.0141933.s007.docx]

**S1 Table. Oligonucleotides used for real time PCR experiments.** The primers name and sequences are reported. The melting temperature (Tm) is indicated in Celsius grade. Primers for *Ikbkg* genes were designed by using Primer3 software (Untergasser A, *et al*. Primer3Plus, an enhanced web interface to Primer3. *Nucl. Acids Res.* 2007 35: W71-4). Primer sequences for *Gapdh* gene were recovered from PrimerBank repository (Spandidos A, *et* al. PrimerBank: a resource of human and mouse PCR primer pairs for gene expression detection and quantification. *Nucl. Acids Res*. 2010 38: D792-9).

| **Primer Name** | **Sequence** | **Tm** |
| --- | --- | --- |
| *Ikbkg fw2* | 5’-GAG GCC CTG GTA GCC AAA C-3’ | 60 |
| *Ikbkg rev2* | 5’-ATG GCA GCC AAC TTT CAG CTT-3’ |  |
| *Tnfaip3 Fw3* | 5’-GAA AAC AAG GGC TTT TGC ACT CT-3’ | 60 |
| *Tnfaip3 Rv3* | 5’-CAG GCA CGG GAC ATT GTT CT-3’ |  |
| *Gapdh PB1 fw* | 5’-AGG TCG GTG TGA ACG GAT TTG-3’ | 60 |
| *Gapdh PB1 rev* | 5’-TGT AGA CCA TGT AGT TGA GGT CA-3’ |  |
