# Supplementary material for: Simultaneous Overexpression of Functional Human HO-1, E5NT and ENTPD1 Protects Murine Fibroblasts against TNF-α-Induced Injury In Vitro
Source: PLoS One. 2015 Oct 29;10(10):e0141933. doi: 10.1371/journal.pone.0141933 (PMC4626094; doi:10.1371/journal.pone.0141933)
Supplement: S2 Table — Genes resulted differently modulated between pCX-TRI-2A-transfected cells and Ctrl cells are listed in table. The genes name and a brief description of the related proteins function are reported. Values are expressed as fold difference in gene expression by comparing each cell lines treated with TNF-α 50 + hemin 20 μM + ATP 200 μM to the respective untreated cells. (DOCX) [file pone.0141933.s008.docx]

**S2 Table. TNF-α pathway-related genes differently modulated between pCX-TRI-2A-transfected cells and Ctrl cells.** Genes resulted differently modulated between pCX-TRI-2A-transfected cells and Ctrl cells are listed in table. The genes name and a brief description of the related proteins function are reported. Values are expressed as fold difference in gene expression by comparing each cell lines treated with TNF-α 50 + hemin 20 μM + ATP 200 μM to the respective untreated cells.

| Genes | Description | Fold up- or down-regulation  TNF-α+HEM+ATP treated cells /  Untreated cells | |
| --- | --- | --- | --- |
|  |  | Ctrl | pCX-TRI-2A |
| *Cd27* | Member of the TNF-receptor superfamily. This receptor is required for generation and long-term maintenance of T cell immunity and it transduces signals that lead to the activation of Nf-kB and MAPK8/JNK. | -4.73 | -3.65 |
| *Tnfrsf11b* | Member of the TNF-receptor superfamily. This protein is an osteoblast-secreted decoy receptor that functions as a negative regulator of bone resorption. May act as decoy receptor for TNFSF10/TRAIL and protect against apoptosis. | -6.01 | -4.88 |
| *Tnfrsf11a* | Member of the TNF-receptor superfamily. This receptor and its ligand are important regulators of the interaction between T cells and dendritic It can interact with various TRAF family proteins, through which it induces the activation of NF-kappa B and MAPK8/JNK. | 1.72 | -4.68 |
| *Tnfrsf18* | Member of the TNF-receptor superfamily. The encoded receptor has been shown to have increased expression upon T-cell activation, and it is thought to play a key role in dominant immunological self-tolerance maintained by CD25(+)CD4(+) regulatory T cells. | -8.57 | -5.39 |
| *Tnfsf11* | Member of the TNF-receptor superfamily. Seems to be involved in interactions between activated T-lymphocytes and endothelial cells and in the regulation of T-cell receptor-mediated cell death. Mediated NF-kappa-B activation and may have a role in the regulation of cell apoptosis. | -1.31 | -3.51 |
| *Tnfaip3* | The protein encoded by this gene is a zinc finger protein and ubiqitin-editing enzyme, and has been shown to inhibit NF-kappa B activation as well as TNF-mediated apoptosis. The encoded protein, which has both ubiquitin ligase and deubiquitinase activities, is involved in the cytokine-mediated immune and inflammatory responses. | 18.38 | 24.03 |
| *Tnf* | Multifunctional proinflammatory cytokine that belongs to the tumor necrosis factor (TNF) superfamily. This cytokine is mainly secreted by macrophages. | 4.73 | -1.08 |
| *Ikbkg* | This gene encodes the regulatory subunit of the inhibitor of kappaB kinase (IKK) complex, which activates NF-kappaB resulting in activation of genes involved in inflammation, immunity and cell survival. Could be implicated in NF-kappa-B-mediated protection from cytokine toxicity. | -1.11 | 1.56 |
| *Traf1* | Member of the TNF receptor (TNFR) associated factor (TRAF) protein family. It associate with, and mediate the signal transduction from various receptors of the TNFR superfamily. Adapter molecule that regulates the activation of NF-kappa-B and JNK. | 24.74 | 17.93 |
